# Dose-Dependent and Subset-Specific Regulation of Midbrain Dopaminergic Neuron Differentiation by LEF1-Mediated WNT1/b-Catenin Signaling

**DOI:** 10.3389/fcell.2020.587778

**Published:** 2020-10-26

**Authors:** Parivash Nouri, Sebastian Götz, Benedict Rauser, Martin Irmler, Changgeng Peng, Dietrich Trümbach, Christian Kempny, Carina G. Lechermeier, Agnes Bryniok, Andrea Dlugos, Ellen Euchner, Johannes Beckers, Claude Brodski, Claudia Klümper, Wolfgang Wurst, Nilima Prakash

**Affiliations:** ^1^Laboratory of Applied Genetics and Stem Cell Biology, Department Hamm 2, Hamm-Lippstadt University of Applied Sciences, Hamm, Germany; ^2^Institute of Developmental Genetics, Helmholtz Zentrum München – German Research Center for Environmental Health, Neuherberg, Germany; ^3^Institute of Experimental Genetics, Helmholtz Zentrum München – German Research Center for Environmental Health, Neuherberg, Germany; ^4^Advanced Institute of Translational Medicine, The First Rehabilitation Hospital of Shanghai, Tongji University School of Medicine, Tongji University, Shanghai, China; ^5^Chair of Experimental Genetics, Technical University of Munich, Munich, Germany; ^6^German Center for Diabetes Research, Neuherberg, Germany; ^7^Department of Physiology and Cell Biology, Zlotowski Center for Neuroscience, Faculty of Health Sciences, Ben-Gurion University of the Negev, Beersheba, Israel; ^8^Chair of Developmental Genetics, Helmholtz Zentrum München, Technical University of Munich/Helmholtz Zentrum München, Neuherberg, Germany; ^9^German Center for Neurodegenerative Diseases, Munich, Germany; ^10^Munich Cluster for Systems Neurology (SyNergy), Munich, Germany

**Keywords:** dopamine, nerve cell, Parkinson’s disease, regenerative therapy, mouse

## Abstract

The mesodiencephalic dopaminergic (mdDA) neurons, including the nigrostriatal subset that preferentially degenerates in Parkinson’s Disease (PD), strongly depend on an accurately balanced Wingless-type MMTV integration site family member 1 (WNT1)/beta-catenin signaling pathway during their development. Loss of this pathway abolishes the generation of these neurons, whereas excessive WNT1/b-catenin signaling prevents their correct differentiation. The identity of the cells responding to this pathway in the developing mammalian ventral midbrain (VM) as well as the precise progression of WNT/b-catenin action in these cells are still unknown. We show that strong WNT/b-catenin signaling inhibits the differentiation of WNT/b-catenin-responding mdDA progenitors into PITX3^+^ and TH^+^ mdDA neurons by repressing the *Pitx3* gene in mice. This effect is mediated by RSPO2, a WNT/b-catenin agonist, and lymphoid enhancer binding factor 1 (LEF1), an essential nuclear effector of the WNT/b-catenin pathway, via conserved LEF1/T-cell factor binding sites in the *Pitx3* promoter. LEF1 expression is restricted to a caudolateral mdDA progenitor subset that preferentially responds to WNT/b-catenin signaling and gives rise to a fraction of all mdDA neurons. Our data indicate that an attenuation of WNT/b-catenin signaling in mdDA progenitors is essential for their correct differentiation into specific mdDA neuron subsets. This is an important consideration for stem cell-based regenerative therapies and *in vitro* models of neuropsychiatric diseases.

## Introduction

The generation of dopamine-synthesizing (dopaminergic, DA) neurons in the mouse midbrain and from human and murine pluripotent stem cells (PSCs) crucially relies on signal transduction by the Wingless-type MMTV integration site family member 1 (WNT1)/beta-catenin (b-catenin, *Ctnnb1*) pathway (Joksimovic and Awatramani, 2014; Brodski et al., 2019). These neurons comprise the Substantia nigra pars compacta (SNc) subset that preferentially degenerates in PD, and the ventral tegmental area (VTA) subpopulation implicated in a variety of human neuropsychiatric disorders, including schizophrenia and addiction (Schultz, 2007; Lees et al., 2009).

The lipid-modified glycoprotein WNT1 is expressed in a bilateral domain within the ventral midline, the floor plate (FP), of the developing midbrain and caudal diencephalon in mammals (Prakash et al., 2006; Brown et al., 2011; Mesman et al., 2014; La Manno et al., 2016). This *Wnt1*^+^ domain comprises only a subset of the mesodiencephalic dopaminergic (mdDA) progenitors located in the ventricular/subventricular zone (VZ/SVZ) of the mesodiencephalic FP (Brown et al., 2011). Yet, WNT1/b-catenin signaling appears to control overall the proliferation of mdDA progenitors and neurogenesis of mdDA precursors expressing the transcription factors (TFs) LMX1A and OTX2, and the aldehyde dehydrogenase ALDH1A1 (AHD2, RALDH1; Joksimovic and Awatramani, 2014; Wurst and Prakash, 2014). However, the participation of WNT1/b-catenin signaling in the subsequent differentiation of these mdDA precursors into mature neurons expressing the full complement of mdDA-specific markers, such as the TFs PITX3 and NURR1 (NR4A2), tyrosine hydroxylase (TH, the rate-limiting enzyme for DA synthesis), dopamine transporter (DAT, SLC6A3), and other proteins implicated in DA metabolism, storage and neurotransmission, remains unclear (Arenas, 2014; Wurst and Prakash, 2014).

A hallmark of the “canonical” WNT/b-catenin signaling pathway is the cytoplasmic accumulation of unphosphorylated (stabilized) b-catenin upon binding of the WNT ligand to its receptor/co-receptor complex on the cell surface, and the subsequent translocation of stabilized b-catenin into the cell nucleus. In the nucleus, b-catenin activates the DNA-bound lymphoid enhancer binding factor 1 (LEF1)/T-cell factors (TCFs) and initiates the transcription of WNT target genes (Nusse and Clevers, 2017). *Ctnnb1*, like *Wnt1*, are necessary for the correct generation in particular of caudally positioned (VTA) mdDA neurons (Prakash et al., 2006; Joksimovic et al., 2009; Tang et al., 2009; Chilov et al., 2010; Andersson et al., 2013; Yang et al., 2013). Overexpression of *Wnt1* or stabilized b-catenin also results in reduced mdDA neuron numbers, especially in the rostral (SNc) domain (Chilov et al., 2010; Tang et al., 2010; Joksimovic et al., 2012; Nouri et al., 2015). These paradoxical findings suggest that WNT1/b-catenin signaling must be tightly balanced in the mdDA progenitors to ensure their proper differentiation into mature mdDA neurons. They also suggest that mdDA progenitors exhibit an unequal responsiveness to this signaling pathway, which contributes to the molecular heterogeneity of different clinically relevant mdDA neuron subsets (Bodea and Blaess, 2015; Fu et al., 2016).

We addressed these issues by a detailed transcriptome analysis of the WNT/b-catenin-responding domain in a transgenic reporter (*BAT-gal*) mouse of “canonical” WNT/b-catenin signaling. This analysis disclosed two components of this pathway, the potent agonist R-spondin 2 (RSPO2) and the nuclear effector LEF1 (Nusse and Clevers, 2017). Both factors are expressed in only a subset of all mdDA progenitors and precursors. We show that high levels of WNT1/b-catenin signaling in these cells, mediated by RSPO2 and LEF1, suppress their differentiation into mature mdDA neurons. This is achieved through direct binding of LEF1 to conserved WNT-responsive elements (*WREs*) in the murine *Pitx3* promoter and WNT/b-catenin-mediated repression of this promoter. Accordingly, only a caudolateral subset of the mdDA progenitors and precursors responded to WNT1/b-catenin signaling throughout mouse embryonic development. Our results provide a mechanistic explanation *in vitro* for the subset-specific effects and the need of a tight regulation of WNT1/b-catenin signaling during mdDA neuron development. These findings might be explored in future applications of PSC-based protocols for the derivation of specific mdDA neuron subsets *in vitro*.

## Materials and Methods

### Mice

Generation of *BAT-gal, TOPGAL*, and *Lef1*^+/−^ mice (*Mus musculus*) was reported previously (van Genderen et al., 1994; DasGupta and Fuchs, 1999; Maretto et al., 2003). These mice were PCR-genotyped using the *LacZ* and *Lef1* primer pairs listed in Supplementary Table 1. *BAT-gal* and *Lef1*^+ /−^ mice were kept as brother-sister matings on mixed (*B6D2F1* or *129S2/SvPas* x *C57BL/6* for *BAT-gal* and *Lef1*^+ /−^, respectively) or *CD-1* (*TOPGAL*) genetic backgrounds. Generation and genotyping of *Wnt1*^+ /−^ and *En1*^+/*Wnt1*^ mice were described by McMahon and Bradley (1990) and Panhuysen et al. (2004). These mice were kept on a *C57BL/6J* genetic background for more than 10 generations. *CD-1* and *C57BL/6J* mice were provided by the Transgenic Unit, Helmholtz Zentrum München. Heterozygote/hemizygote *Wnt1*^+ /−^; *BAT-gal^+^* and *Lef1*^+ /−^; *BAT-gal^+^* mice were generated by crossing *Wnt1*^+ /−^ and *Lef1*^+ /−^ mice with the transgenic *BAT-gal* mouse line. Homozygote/hemizygote *Wnt1*^–/–^; *BAT-gal^+^* and *Lef1*^–/–^; *BAT-gal^+^* embryos were obtained from intercrosses of heterozygote/hemizygote *Wnt1*^+ /−^ or *Lef1*^+ /−^; *BAT-gal^+^* and *Wnt1*^+ /−^ or *Lef1*^+ /−^; *BAT-gal^–^* mice. Pregnant dams were killed by CO_2_ asphyxiation or cervical dislocation. Collection of embryonic stages of either sex was done from timed-pregnant females; noon of the day of vaginal plug detection was designated as E0.5. This study was carried out in strict accordance with the recommendations in the European Union Directive 2010/63/EU and the Guide for the Care and Use of Laboratory Animals of the Federal Republic of Germany (German Animal Protection Law). The protocol was approved by the Institutional Animal Care and Use Committee (Committee for Animal Experiments and Laboratory Animal Facility) of the Helmholtz Zentrum München. All efforts were made to minimize suffering.

### Laser-Microdissection

The RNA integrity-preserving tissue staining protocol by Brown and Smith (2009) for subsequent Laser-Microdissection (LMD) and microarray analysis was modified as follows: E12.5 *BAT-gal* embryo heads were dissected in ice-cold PBS, fixed for 2 h in 4% PFA at 4°C, incubated in 20% sucrose overnight, embedded in Neg-50 (Thermo Fisher Scientific), frozen in 2-methylbutane on dry ice and stored at −80°C. Consecutive coronal cryosections (20 μm) from the caudal forebrain (diencephalon) and midbrain were mounted on POL frame slides (Leica Microsystems) and stored at −80°C. Mounted sections were post-fixed in acetone at −20°C, dried at 40°C for 1 min, washed twice with cold 2 M NaCl PBS, permeabilized with 0.25% Triton X-100 in 2 M NaCl PBS for 2 h, incubated with primary antibodies [rabbit anti-PITX3 1:100; Thermo Fisher Scientific 38-2850; chicken anti-b-Galactosidase (b-GAL) 1:1000; abcam ab9361] overnight and secondary antibodies (AlexaFluor 594 donkey anti-rabbit IgG and AlexaFluor 488 donkey anti-chicken IgG; Thermo Fisher Scientific; each diluted 1:250 in permeabilization solution at 4°C) for 2 h, washed three times with 2 M NaCl PBS and stored in this solution at 4°C. Immediately before LMD, tissue sections were dehydrated in 70% and 100% ethanol (2 min each), the ROIs were outlined under visual inspection, and tissue fragments corresponding to the WNT/b-catenin-responding mdDA domain or the lateral non-WNT/b-catenin-responding, non-mdDA domain were isolated with an LMD6000 system (Leica Microsystems) and stored at −80°C.

### Microarray Analysis

Total RNA was isolated from the laser-microdissected tissue fragments using the RNeasy FFPE kit (Qiagen), and RNA quality and quantity were determined with the 2100 Bioanalyzer and RNA 6000 Pico kit (Agilent Technologies) following the instructions of the manufacturers. Total RNA (40 ng) from each tissue fraction was amplified with the Ovation PicoSL WTA System V2 in combination with the Encore Biotin Module (Tecan). Amplified cDNAs were hybridized on Mouse Gene 1.0 ST arrays (Affymetrix), and staining and scanning were done according to the Affymetrix expression protocol including minor modifications as suggested in the Encore Biotin protocol. Expression console (Affymetrix) was used for quality control and to obtain annotated normalized RMA gene-level data (standard settings including sketch-quantile normalization). Fluorescence-activated cell sorting (FACS) and microarray analysis of E13.5/E14.5 *Pitx3*^*GFP/+*^ mdDA neurons were reported by Fukusumi et al. (2015).

### Radioactive *in situ* Hybridization

Serial paraffin sections (8 μm) were hybridized with radioactive ([α-^35^S]UTP, Perkin-Elmer) riboprobes as described previously (Brodski et al., 2003; Fischer et al., 2007). *In Situ* Hybridization (ISH) probes used were *Wnt1* (Fischer et al., 2007); *Lmx1a*, *Lef1*, and *Tcf7l2* (Zhang et al., 2015); *Pitx3* (Brodski et al., 2003); *Nurr1* and *Aldh1a1* (Stuebner et al., 2010); *Dkk3* (Monaghan et al., 1999); or newly cloned [*Apcdd1*, *Cck*, *Fgf14*, *Pbx1*, *Rspo2*, *Smarca1*, *Sulf1*, *Sulf2*, *Tcf7*, and *Tcf7l1*] into pCRII-TOPO vector (Thermo Fisher Scientific) by PCR from E10.5 or E12.5 *CD-1* whole embryo or head cDNA using the primer pairs listed in Supplementary Table 1. Images were taken with an Axioplan 2 microscope, AxioCam MRc camera and Axiovision 4.6 software (Zeiss) using bright-field and dark-field optics, and processed with Adobe Photoshop CS5 or CC. Dark-field and bright-field images were pseudocolored in some cases, as indicated in the figure legends.

### Immunohistochemistry/Immunocytochemistry

Immunohistochemistry (IHC) on serial 8 μm paraffin sections and Immunocytochemistry (ICC) on cultured cells were performed as reported previously (Puelles et al., 2004; Peng et al., 2011). Polyclonal antisera used were chicken anti-b-GAL (1:2000; abcam ab9361); rabbit anti-PITX3 (1:300; Thermo Fisher Scientific 38-2850), anti-SOX6 (1:500; abcam ab30455), anti-TH (1:150; Merck Millipore AB152), and anti-LMX1A (1:1000; Merck Millipore ab10533); and goat anti-OTX2 (1:20; R&D Systems AF1979). Monoclonal antibodies (mAbs) used were rabbit anti-LEF1 (C12A5; 1:100; Cell Signaling Technology 2230); mouse anti-TH (1:600; Merck Millipore MAB318); and rat anti-DAT (1:500; Merck Millipore MAB369). The guinea pig anti-SOX6 antibody [1:750; (Panman et al., 2014)] was a kind gift from Dr. J. Muhr (Karolinska Institute, Stockholm, Sweden). All secondary antibodies were fluorescently labeled (AlexaFluor 488/555/594, 1:500; Thermo Fisher Scientific). Cultured cells were counterstained with 4′,6-diamidino-2-phenylindole (DAPI). Fluorescent images were taken with an Axiovert 200 M microscope, AxioCam HRc camera and Axiovision 4.6 software (Zeiss), Olympus IX81 confocal laser-scanning microscope and Fluoview FV1000 2.0b software (Olympus), or DMi8 inverted microscope, DFC9000 GT camera and LAS X 3.0.0 software (Leica Microsystems), and processed with Adobe Photoshop CS5 or CC (Adobe Systems).

### VM Primary Cultures and RSPO2/EdU Treatments

Ventral midbrain (VM) primary cultures were prepared from E11.5 *CD-1* mouse embryos as described by Pruszak et al. (2009) with minor modifications. Briefly, VM tissues were trypsinized for 5 min at 37°C, and 2 × 10^5^ cells/well were plated in a 24-well plate on poly-D-lysine (Merck)-coated coverslips in DMEM/F-12 medium supplemented with 1% penicillin/streptomycin (both from Thermo Fisher Scientific), 10% fetal bovine serum (FBS, PAN Biotech), and 100 μM ascorbic acid (Merck). 1 day (24 h) after plating *in vitro* (1 DIV), cells were incubated in expansion medium for 1 day (2 DIV) and then switched to differentiation medium (Pruszak et al., 2009) for the remaining culture time (3–10 DIV). Concomitant with the change to differentiation medium at 3 DIV, VM primary cells were treated with 2.5–100 ng/ml recombinant human RSPO2 protein [R&D Systems 3266-RS; in 0.1% bovine serum albumin (BSA, Merck)] or 20 μg/ml BSA (0 ng/ml RSPO2 control) for 7 days (10 DIV). After 3 days (at 6 DIV), the medium was replaced by fresh differentiation medium containing different RSPO2 concentrations or BSA. Cells were fixed and processed after 10 DIV for the detection of TH and PITX3 by ICC. In some cases, VM primary cells were incubated with 10 μM 5-ethynyl-2′-deoxyuridine (EdU; Thermo Fisher Scientific) for 2 h before fixation after 1 day (4 DIV) or 3 days (6 DIV) of RSPO2-treatment. Incorporated EdU was detected using the Click-iT EdU AlexaFluor 488 Imaging Kit (Thermo Fisher Scientific) according to the manufacturer’s instructions.

### Cell Countings and Unbiased Stereology

For ICC on VM primary cells, TH and PITX3 single- and double-labeled cells were counted on 1-2 coverslips/treatment/experiment. EdU- per DAPI-positive cells were determined using a CellInsight NXT high content screening platform (50 pictures at 10x magnification) and CellInsight NXT software (Thermo Fisher Scientific). For IHC, LMX1A, SOX6, PITX3, or OTX2 single- and b-GAL/LMX1A, b-GAL/SOX6, b-GAL/PITX3, or b-GAL/OTX2 double-positive cells in the E10.5, E12.5, E14.5, and E17.5 or E18.5 *BAT-gal* VM, respectively, were counted bilaterally on every fourth (E10.5, E12.5), sixth (E14.5), or eighth (E17.5/E18.5) serial coronal midbrain section using Stereo Investigator 5.05.4 (MBF Bioscience) or LAS X 3.0.0 and ImageJ 1.52a (NIH/United States) software. Consecutive serial sections stained for b-GAL and TH were used to outline the mdDA domain in these cases. TH and PITX3 single-labeled cells in the wild-type (*wt*) and *Lef1*^–/–^ VM were evaluated bilaterally by the optical fractionator method on every fourth (E12.5) or eighth (E17.5) serial coronal midbrain section using Stereo Investigator 5.05.4 software. Cell numbers were averaged for each genotype, stage, region or treatment and subjected to tests for the estimation of statistical significance.

### Western Blot

Ventral midbrain and dorsal midbrain (DM) tissues from E12.5 *CD-1* mouse embryos were dissected in ice-cold PBS and homogenized in RIPA buffer [50 mM Tris–HCl pH7.4, 150 mM NaCl, 1% Triton X-100, 0.5% Sodium Deoxycholate, 0.1% SDS, 3 mM EDTA, and protease inhibitors (complete Mini; Merck)]. Total proteins (20 μg per sample, determined with Pierce BCA Protein Assay; Thermo Fisher Scientific) were separated by SDS-PAGE, blotted on Hybond-LFP membranes (Merck) and stripped as described by Fischer et al. (2011). Blots were probed with two different rabbit anti-LEF1 mAbs [C12A5 (#2230) and C18A7 (#2286); 1:1000, Cell Signaling Technology], mouse anti-b-actin (ACTB) mAb (1:5000; abcam ab6276) and HRP-conjugated goat-anti-rabbit (Dianova 111-035-003) and goat-anti-mouse (Dianova 115-035-003) secondary antibodies (each 1:5000), developed in ECL substrate and exposed to Hyperfilm ECL (Merck).

### Prediction of LEF1/TCF Binding Sites/*WREs* in the *Pitx3* Promoter Region

Promoter sequences for *Pitx3* genes were derived from the ElDorado genome database [Genomatix version 12-2016 (NCBI build 38), Germany]. Orthologous promoter sequences from different mammalian species of the Genomatix homology group (mouse, rat, human, chimp, and rhesus monkey) were analyzed using the MatInspector program (Genomatix, Matrix Family Library Version 10.0) to identify potential LEF1/TCF Binding Sites (BSs)/*WREs* and to extract matrix similarities. Conserved BSs/*WREs* were determined with the DiAlign TF program (Genomatix) and by searching for common LEF1/TCF BSs/*WREs* occurring at the same position in (aligned) orthologous *Pitx3* promoter sequences. The *Pitx3* promoter regions were defined as ∼2000 bp upstream, including the proximal region, and ∼500 bp downstream of the transcription start site (TSS).

### Chromatin Immunoprecipitation-PCR

Ventral midbrain tissues were dissected from E11.5 *CD-1* embryos, and Chromatin Immunoprecipitation (ChIP) was done using the EZ-ChIP kit (Merck Millipore 17-371) according to the manufacturer’s instructions. Sheared genomic DNA fragments after sonication with a Sonopuls Sonicator (Bandelin; output setting 60%, 10 times 10 s pulses with 10 s incubation on ice between pulses) were enriched between 200–800 bp. Two different rabbit anti-LEF1 mAbs [C12A5 (#2230) and C18A7 (#2286); 1 μg each, Cell Signaling Technology], anti-RNA Polymerase II antibody (Merck Millipore) or rabbit IgG (rIgG; Jackson ImmunoResearch 78057) were used for ChIP. Purified DNA served as template for PCR amplification of *Pitx3* genomic fragments with the six primer pairs listed in Supplementary Table 1.

### Transfections, Luciferase Reporter Assays and Site-Directed Mutagenesis

HEK-293, COS-7, and SH-SY5Y cells were derived from cell lines available at Helmholtz Zentrum München. HEK-293 cells were STR-genotyped and tested for mycoplasma contamination at Hamm-Lippstadt University of Applied Sciences. HEK-293 and COS-7 cells were kept in DMEM, 10% fetal calf serum (FCS) or FBS (PAN Biotech) and 2 mM L-glutamine; SH-SY5Y cells were kept in DMEM/F-12 (all from Thermo Fisher Scientific) and 15% FCS at 37°C and 5% CO_2_. HEK-293, COS-7 and SH-SY5Y cells (5 × 10^4^ cells/well of a 24-well plate) were transfected with 200 ng/well *BAT-gal* plasmid [Addgene plasmid 20889 (Maretto et al., 2003)] using Lipofectamine 2000 and fixed after 24 h for ICC. Using Lipofectamine 2000 or TurboFect Transfection Reagent (Thermo Fisher Scientific), 5 × 10^4^ or 2 × 10^5^ HEK-293 cells/well of a 24-well plate were co-transfected with 200 ng/well of each reporter construct [*Super8xTOPFlash* or *Super8xFOPFlash* (Lie et al., 2005), or a wild-type or mutagenized 2514 bp mouse *Pitx3* promoter fragment (-2425 to +89) in *pGL3* basic vector (Peng et al., 2011)], 0.1 ng/well *pRL-SV40* (Promega) internal transfection control and *pcDNA3.1* (Thermo Fisher Scientific) “empty” vector to adjust for DNA content; alone or together with 200 ng/well or varying amounts of stabilized *pcDNA3-S33Y b-catenin* [Addgene plasmid 19286, (Kolligs et al., 1999)] with or without 200 ng/well or varying amounts of human *LEF1* cDNA (Waterman et al., 1991), and vice versa, 166 ng/well *pMES-Lmx1a-IRES-eGFP* (Klafke et al., 2016), or 353.8 ng/well *LMX1A* siRNA (#144, Supplementary Table 1). The human *LEF1* cDNA comprises the 399 aa ORF of full-length human LEF1 protein (including the N-terminal b-catenin binding domain) and was confirmed by sequencing (SEQLAB GmbH, Göttingen, Germany). Cells were treated with BSA (20 μg/ml), recombinant human WNT1 (100 ng/ml; PeproTech 120-17), or RSPO2 protein (2.5–250 ng/ml) 1 h post-transfection in some cases, and lysed in Passive Lysis Buffer (Promega) after 24 h. Firefly/Renilla luciferase luminescence was measured in a Centro LB 960 luminometer (Berthold Technologies) or SpectraMax i3x multimode plate reader (Molecular Devices) using the Dual-Luciferase Reporter Assay (Promega) according to the instructions of the manufacturers. Firefly luminescence was normalized to Renilla luminescence for each well, and these values were normalized to the control condition of each experiment, as indicated in the figure legends. Site-directed mutagenesis of the three most conserved and proximal (relative to the TSS) LEF1/TCF BSs/*WREs* (*WRE 1-3*) predicted in the ∼2.5 kb mouse *Pitx3* promoter fragment was done with the mutagenic primers listed in Supplementary Table 1 and the QuikChange Lightning Multi Site-Directed Mutagenesis Kit (Agilent Technologies) according to the manufacturer’s instructions. Mutant *Pitx3* promoter fragments were confirmed by sequencing (SEQLAB GmbH).

### RT-PCR

Total RNA was isolated from HEK-293, COS-7 and SH-SY5Y cells with Trizol Reagent (Thermo Fisher Scientific) or RNeasy Mini Kit (Qiagen), including a DNAse I treatment. 1 μg total RNA was reverse transcribed using Advantage RT-for-PCR Kit (Takara Bio) or Quantitect Reverse Transcription Kit (Qiagen) according to the instructions of the manufacturers. 1.0 or 2.5 μl cDNA were amplified by PCR using the intron-spanning primers and conditions listed in Supplementary Table 1. All assays included negative controls.

### Experimental Design and Statistical Analyses

The LMD and microarray data are duplicates (technical replicates) of laser-microdissected tissue pools from five E12.5 *BAT-gal^+^* embryos. For ICC on cultured cells, five or three independent experiments were evaluated in each case, as indicated in the figure legend. For ChIP-PCR experiments, data are from five independent ChIP experiments (biological replicates), and PCR assays were repeated three times for each experiment (technical replicates). The luciferase reporter assays were performed in triplicates (technical replicates), and data are derived from three to seven independent experiments (biological replicates), as indicated in the figure legends. For IHC and ISH, mutant [homozygote (*-/-*) or hemizygote *BAT-gal^+^*] embryos were compared to their *wt* [homozygote (*+/+*) and heterozygote (*+/-*) or hemizygote *BAT-gal^–^*] littermates and at least 3 embryos from at least 2 different litters were analyzed for each probe, genotype and stage, unless stated otherwise. For microarrays, statistical analyses were performed with the statistical programming environment R (R Core Team, 2013) implemented in CARMAweb (Rainer et al., 2006). Gene-wise testing for differential expression was done using the limma paired *t*-test and Benjamini-Hochberg multiple testing correction [false discovery rate (FDR) < 10%]. Heat maps were generated with R. Genes associated with the WNT receptor signaling pathway were obtained from canonical pathways and pathway associations from GePS software (Genomatix). All other values are mean ± s.e.m. For comparison of two groups in the unbiased stereological quantifications, the Welsh *t*-test for unequal variances was applied using the R software with the nlme package (R Core Team, 2013). All other statistical analyses were done with GraphPad Prism 5/8 or IBM SPSS Statistics 25 software. The effects of experimental factors with more than two levels and the effect of two or more experimental factors were analyzed with one-way or two-way ANOVA, depending on the number of factors. For quantifications of single- and double-positive VM primary cells/coverslip, two-way ANOVA for repeated measurements followed by Bonferroni’s posttests was applied. Kruskal–Wallis with Dunn’s multiple comparisons *post hoc* tests and two-tailed unpaired *t*-tests were applied for comparison of individual factors, and one-way ANOVA followed by Bonferroni’s multiple comparisons *post hoc* tests were applied for comparison of all factors in an experiment. Luciferase assays with the *wt* and mutant mouse *Pitx3* promoter and different WNT/b-catenin signaling conditions were analyzed by Wilcoxon–Mann–Whitney test.

## Results

### Transcriptome Profiling of the WNT/b-Catenin-Responding mdDA Domain in the E12.5 *BAT-gal^+^* Mouse VM

**FIGURE 1:**
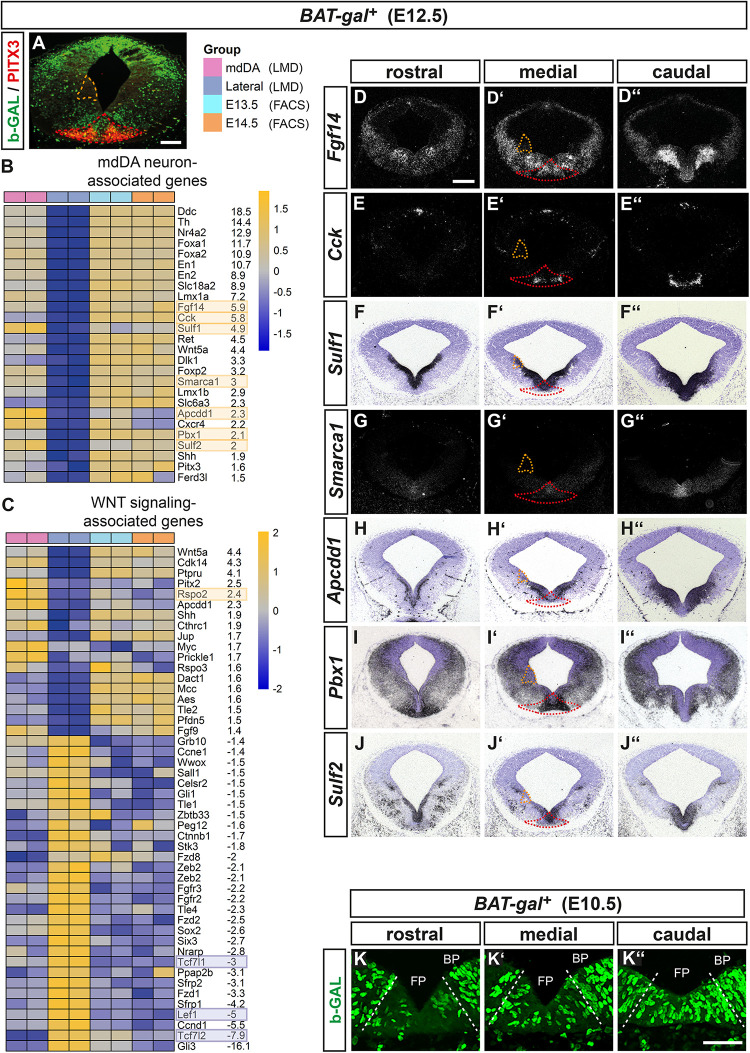
Transcriptome profiling of the WNT/b-catenin-responding mdDA domain in the E12.5 *BAT-gal^+^* mouse VM. **(A)** Representative coronal section at medial midbrain level of an E12.5 *BAT-gal^+^* embryo, immunostained with antibodies against b-GAL (green), and PITX3 (red). Red broken line delimits the WNT/b-catenin-responding mdDA domain, orange broken line delimits the lateral non-WNT/b-catenin-responding non-mdDA domain, which were laser-microdissected and used for the comparative microarray-based transcriptome analysis. Right side depicts the legend for the heat map analysis of differentially regulated genes (FDR < 10%) in the laser-microdissected (LMD) WNT/b-catenin-responding mdDA domain (mdDA) relative to the lateral non-WNT/b-catenin-responding non-mdDA domain (Lateral). Data of FACS-sorted GFP-expressing mdDA neurons from E13.5 and E14.5 *Pitx3*^*GFP/+*^ embryos (E13.5, E14.5) are shown in addition. Two technical replicates were analyzed for each sample. **(B,C)** Heat maps representing expression levels (yellow: overrepresented; blue: underrepresented) of selected mdDA neuron-associated **(B)** or WNT signaling-associated **(C)** genes in the laser-microdissected WNT/b-catenin-responding mdDA domain relative to the lateral non-WNT/b-catenin-responding non-mdDA domain and FACS-sorted E13.5/E14.5 *Pitx3*^*GFP/+*^ mdDA neurons. Average fold-changes shown are WNT/b-catenin-responding mdDA domain vs. lateral non-WNT/b-catenin-responding non-mdDA domain. Yellow and blue boxes denote the genes whose expression patterns were analyzed *in situ*. **(D–J”)** Representative cresyl violet-stained coronal sections (dorsal top) at different rostrocaudal levels of the *BAT-gal^+^* midbrain at E12.5, hybridized with riboprobes for *Fgf14* **(D–D”)**, *Cck* **(E–E”)**, *Sulf1* **(F–F”)**, *Smarca1* **(G–G”)**, *Apcdd1* **(H–H”)**, *Pbx1* **(I–I”)**, and *Sulf2* **(J–J”)**. **(D–E”,G–G”)**, dark-field images. **(K–K”)** Representative close-up views of the VM on coronal sections (dorsal top) at different rostrocaudal levels of the *BAT-gal^+^* midbrain at E10.5, immunostained for b-GAL (green). Broken white lines delimit the FP from BP. Scale bars: 200 μm **(A,D)**; 50 μm **(K”)**.

To identify downstream effectors and potential targets as well as modulators of WNT/b-catenin signaling in mdDA cells, we laser-microdissected and transcriptome-profiled the WNT/b-catenin-responding mdDA domain in the midgestational (E12.5) mouse embryo. At this embryonic stage, WNT/b-catenin signaling is fully active in the mouse VM (Fukusumi et al., 2015). We used the *BAT-gal* mouse, in which nuclear b-GAL protein is expressed upon activation of a minimal promoter containing seven multimerized LEF1/TCF BSs by a “canonical” (b-catenin-mediated) WNT signal (Maretto et al., 2003). The WNT/b-catenin-responding (b-GAL^+^) mdDA domain was co-labeled with PITX3 for unambiguous identification and isolated from the VM of five E12.5 *BAT-gal^+^* embryos (Figure 1A). A non-WNT/b-catenin-responding and non-mdDA (b-GAL^–^/PITX3^–^) domain at the alar-basal boundary (ABB) was laser-microdissected and profiled in the same experiment to enrich for specific transcripts of the WNT/b-catenin-responding mdDA domain (Figure 1A). This strategy resulted in a strong concordance of the global transcriptome between the laser-microdissected WNT/b-catenin-responding mdDA domain and FACS-sorted *Pitx3*^*GFP/+*^ mdDA precursors/neurons reported previously (Fukusumi et al., 2015; Figures 1B,C and data not shown). As expected, many mdDA- or FP-related transcripts [e.g., *Ddc* (*Aadc*), *Th*, *Nr4a2* (*Nurr1*), *Foxa1*/*2* (*Hnfa*/*b*), *En1*/*2*, *Slc18a2* (*Vmat2*), *Lmx1a*, *Ret*, *Wnt5a*, *Dlk1*, *Lmx1b*, *Slc6a3* (*Dat*), *Cxcr4*, *Shh*, *Pitx3*, and *Ferd3l* (*Nato3*) (Arenas et al., 2015)] were significantly (FDR < 10%) and at least 1.5- up to 18-fold enriched in the WNT/b-catenin-responding mdDA domain relative to the lateral non-WNT/b-catenin-responding and non-mdDA domain (Figure 1B). Notably, far fewer WNT signaling-associated transcripts were enriched in the WNT/b-catenin-responding mdDA domain relative to the lateral domain (Figure 1C).

To validate this approach, we determined the expression pattern of several transcripts overrepresented in the WNT/b-catenin-responding mdDA domain, including genes with confirmed expression in the mouse VM or mdDA domain [*Fgf14* (Wang et al., 2000), cholecystokinin (*Cck*; Jacobs et al., 2011), *Sulf1*/*2* (Ratzka et al., 2010), *Apcdd1* (*Drapc1*; Jukkola et al., 2004)], and function in mdDA neuron development [*Smarca1* (*Snf2l*; Metzakopian et al., 2015), *Pbx1* (Villaescusa et al., 2016)]. All transcripts were detected within the midbrain FP but none (except for *Sulf1*) in the lateral domain at the ABB, thus confirming their selective enrichment in the mouse VM/mdDA domain (Figures 1D–J”). Notably, some genes overrepresented in the WNT/b-catenin-responding mdDA domain were expressed either exclusively or prominently in the mediocaudal VM [*Cck* (Figures 1E–E”), *Smarca1* (Figures 1G–G”), and *Dlk1* [Figure 1C; (Veenvliet and Smidt, 2014)]. This expression pattern suggested that the WNT/b-catenin-responding mdDA cells belong to a mediocaudal progenitor/precursor subset. Indeed, b-GAL^+^ (WNT/b-catenin-responding) cells were detected mostly in the FP of the medial and caudal *BAT-gal^+^* midbrain at E10.5 (Figures 1K–K”).

### RSPO2-Mediated Activation of WNT1/b-Catenin Signaling Inhibits the Differentiation of PITX3^+^ mdDA Neurons *in vitro*

**FIGURE 2:**
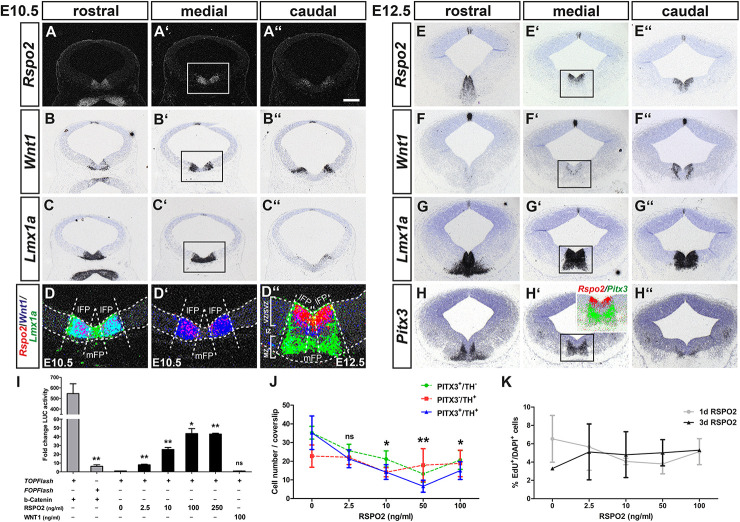
RSPO2-mediated activation of WNT1/b-catenin signaling inhibits the differentiation of PITX3^+^ mdDA neurons *in vitro*. **(A–H”)** Representative coronal overviews **(A–C”,E–H”)** at different rostrocaudal levels of the midbrain (dorsal top) from *CD-1* embryos at E10.5 **(A–D’)** and E12.5 **(D”–H”)**, hybridized with riboprobes for *Rspo2* **(A–A”,E–E”)**, *Wnt1* **(B–B”,F–F”)**, *Lmx1a* **(C–C”,G–G”)**, and *Pitx3* **(H–H”)**. **(A–A”)** dark-field images. (**D–D”**, inset in **H’**), pseudocolored overlays of consecutive medial midbrain sections (higher magnification dark- or bright-field views of boxed areas in **A’–C’** and **E’–H’**, respectively), hybridized with probes for *Rspo2* (red), *Wnt1* (blue), and *Lmx1a* (green; **D–D”**) or *Pitx3* (green; inset in **H’**), overlapping expression domains appear in magenta (red/blue), cyan (blue/green), or yellow (red/green). Broken white lines in **(D–D”)** outline the neuroepithelium and delimit lFP from mFP; white lines in **(D”)** delimit VZ/SVZ from IZ and MZ. **(I)** Fold change of luciferase (LUC) activity in HEK-293 cells (relative to only BSA-treated cells, set as 1) after transfection with *TOPFlash* or *FOPFlash* reporter and *S33Y-b-catenin*, or *TOPFlash* reporter and treatment with 10 μg BSA, increasing amounts of RSPO2 (2.5–250 ng/ml) or 100 ng/ml WNT1 protein [*n* = 3 independent experiments; statistical testing for significance between *TOPFlash* and *FOPFlash* + b-catenin (*t* = 5.8, df = 4, *P* = 0.0044, and unpaired *t*-test) or in relation to only BSA-treated cells]. **(J)** Quantification of PITX3^+^/TH^–^ (green line), PITX3^–^/TH^+^ (red line), and PITX3^+^/TH^+^ (blue line) cells after treatment of VM primary cultures with BSA (10 μg) or increasing amounts of RSPO2 protein (2.5–100 ng/ml) for 7 days (10 DIV) [*n* = 5 independent experiments; statistical testing for significance only shown for double-positive cells in relation to BSA-treated cultures in two-way ANOVA for repeated measurements followed by Bonferroni’s posttests; F(4,36) = 6.28, *P* = 0.0024]. **(K)** Quantification of EdU^+^ per DAPI^+^ cells after treatment of VM primary cultures with BSA (10 μg) or increasing amounts of RSPO2 protein (2.5–100 ng/ml) for 1 day (4 DIV, gray line) or 3 days (6 DIV, black line) [*n* = 3 independent experiments; no significant change of EdU^+^/DAPI^+^ cells in relation to only BSA-treated cells in two-way ANOVA for repeated measurements followed by Bonferroni’s posttests; F(4,19) = 0.24]. *P < 0.05; **P < 0.005; and ns, not significant. Scale bar: 200 μm **(A”)**.

Among the genes with known functions in the WNT signaling pathway, *Rspo2* was significantly (FDR < 10%) enriched by approx. 2.4-fold in the WNT/b-catenin-responding mdDA domain (Figure 1C). *Rspo2* is an activator of WNT/b-catenin signaling, regulated by LMX1A, and expressed in the developing mouse VM (Kim et al., 2008; Hoekstra et al., 2013; Gyllborg et al., 2018). At E10.5 and E12.5, *Rspo2* was transcribed in bilateral stripes within the *Lmx1a*^+^ FP, overlapping with the bilateral *Wnt1* expression domain in the lateral FP (lFP) and sparing the medial FP (mFP) in the mouse midbrain (Figures 2A–G”). Interestingly at E12.5, when VZ/SVZ and mantle zone (MZ) are clearly distinguishable (Figure 2D”), a gap was detected between *Rspo2* and *Pitx3* expression (Figures 2E–E”,H–H”; inset in Figure 2H’). These data suggested that *Rspo2* is involved in the activation or enhancement of WNT1-mediated signaling in *Lmx1a*^+^ mdDA progenitors within the VZ/SVZ, but has to be downregulated in these progenitors as soon as they begin to differentiate into postmitotic *Lmx1a*^+^ and *Pitx3*^+^ mdDA precursors and neurons in the mouse VM.

To test this hypothesis, we first determined whether increasing amounts of soluble RSPO2 protein (2.5–250 ng/ml) activated a *TOPFlash* reporter for “canonical” WNT/b-catenin signaling in a dose-dependent manner in WNT/b-catenin-responding HEK-293 cells (see Supplementary Figure 2). This was indeed the case, peaking at 100 ng/ml RSPO2 with a 44 ± 5.6-fold increase of luciferase activity compared to untreated cells (Figure 2I). RSPO2-mediated activation of WNT/b-catenin signaling was not as effective as a constitutively active (stabilized) *S33Y-b-catenin* (545 ± 93-fold increase), but clearly recognizable in contrast to soluble WNT1 protein, which did not activate the *TOPFlash* reporter at a concentration normally promoting mdDA differentiation (Joksimovic et al., 2009; Fukusumi et al., 2015; Figure 2I). Next, we treated differentiating VM primary cells with increasing RSPO2 concentrations *in vitro*. Increasing amounts of RSPO2 led to a dose-dependent and significant decrease of PITX3^+^/TH^+^ double-positive cells (maturing mdDA neurons) and PITX3^+^/TH^–^ single-positive mdDA precursors in these cultures, peaking at 50 ng/ml RSPO2 (Figure 2J and Supplementary Figure 1). Notably, the ICC signal intensity of nuclear PITX3 protein appeared to be reduced in cells treated with increasing RSPO2 concentrations (Supplementary Figure 1). The PITX3^–^/TH^+^ single-positive cell numbers, by contrast, remained almost constant in the RSPO2-treated cultures (Figure 2J and Supplementary Figure 1). The reduced differentiation of PITX3^+^/TH^+^ mdDA neurons by increasing amounts of RSPO2 was not due to an increase in proliferation of VM progenitors (EdU^+^ cells) in these cultures (Figure 2K). Our results thus suggest that RSPO2 activates or enhances WNT1/b-catenin signaling in LMX1A^+^ mdDA progenitors and at the same time inhibits their (premature) differentiation into PITX3^+^/TH^+^ mdDA neurons. Transcription of *Rspo2* must therefore be downregulated at the transition from a PITX3^–^ mdDA progenitor to a PITX3^+^ mdDA precursor in the IZ of the murine VM.

### WNT1/b-Catenin-Responsiveness in the Mouse VM Is Mediated by the Activating LEF/TCF Family Member LEF1

**FIGURE 3:**
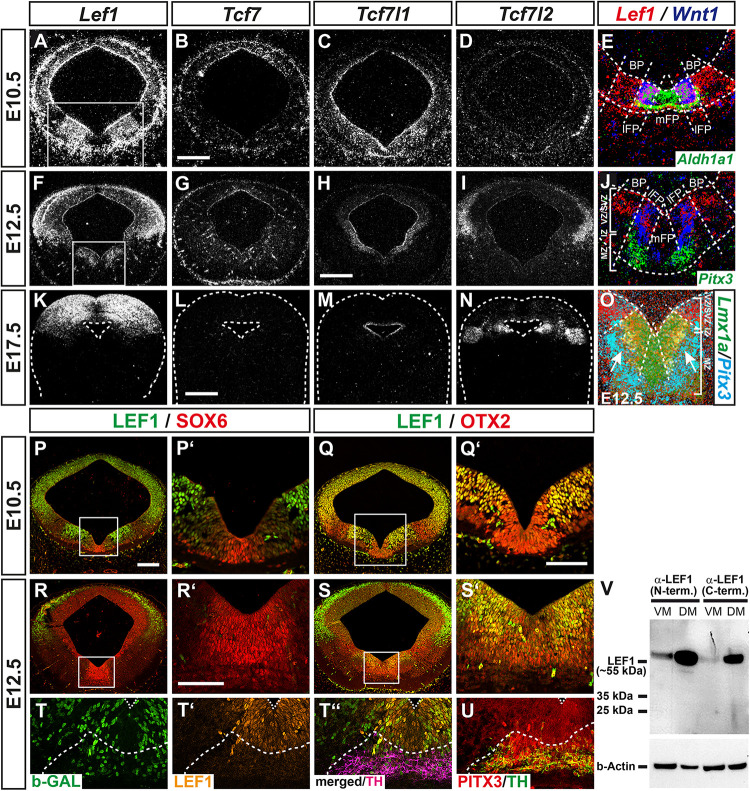
WNT1/b-catenin-responsiveness in the mouse VM is mediated by the activating LEF/TCF family member LEF1. **(A–U)** Representative overviews **(A–D,F–I,K–N,P–S)** and close-up views of the VM **(E,J,O,P’–S’)** on coronal sections (dorsal top) at medial **(A–Q’,S–U)** and rostral **(R,R’)** midbrain levels from *CD-1* **(A–S’)** and *BAT-gal^+^* **(T–U)** embryos at E10.5 **(A–E,P–Q’)**, E12.5 **(F–J,O,R–U)** and E17.5 **(K–N)**, hybridized with riboprobes for *Lef1* **(A,F,K)**, *Tcf7* **(B,G,L)**, *Tcf7l1* **(C,H,M)** and *Tcf7l2* **(D,I,N)**, or immunostained with antibodies against LEF1 (green, **P–S’**; orange, **T’,T”**), SOX6 (red; **P,P’,R,R’**), OTX2 (red; **Q,Q’,S,S’**), b-GAL (green; **T,T”**), TH (magenta, **T”**; green, **U**) and PITX3 (red; **U**; same section in **T–T”**, adjacent section in **U**). **(A–O)**, dark-field images; **(E,J,O)**, pseudocolored overlays of consecutive sections (higher magnifications of boxed areas in **A** and **F**, respectively), hybridized with probes for *Lef1* (red), *Wnt1* (blue), and *Aldh1a1* (green; **E**), *Pitx3* (green, **J**; cyan, **O**), or *Lmx1a* (green; **O**); overlapping expression domains appear in magenta (red/blue), cyan (blue/green), yellow (red/green), or white (red/blue/green). Broken white lines outline the neuroepithelium **(E,J,K–O)** and delimit BP from lFP and mFP **(E,J,O)** or TH^+^/PITX3^+^ domain from LEF1^+^ domain **(T–U)**; white lines in **(J,O)** delimit the VZ/SVZ, IZ, and MZ. **(V)** Western blot analysis of LEF1 expression in VM and DM tissues of E12.5 *CD-1* mouse embryos, using two different LEF1 antibodies against the N- (C12A5) or C-terminus (C18A7) of LEF1. Detection of b-actin was used as loading control. The position of the ∼55 kDa full-length (activating) LEF1 isoform and smaller protein bands are indicated to the left. Scale bars: 200 μm **(B,H)**; 500 μm **(L)**; 100 μm **(P)**; and 50 μm **(Q’,R’)**.

Wingless-type MMTV integration site family member 1 (WNT1) and RSPO2 are secreted molecules, whose activity might not be confined to the *Wnt1*- and *Rspo2*-expressing cells in the VZ/SVZ of the lFP. Therefore, we focused on those genes that might identify the WNT/b-catenin-responding mdDA progenitors and precursors in the mouse VM. Strong candidates in this context were the DNA-binding proteins of the LEF1/TCF family. They are the last and crucial component that mediate the activating or repressing effects of this signaling pathway on WNT target gene transcription upon binding of the co-activator b-catenin or of other co-repressors, respectively (Nusse and Clevers, 2017). Three members of this family, *Lef1*, *Tcf7l1* (*Tcf3*), and *Tcf7l2* (*Tcf4*), were overrepresented in the non-WNT/b-catenin-responding and non-mdDA domain in the lateral midbrain relative to the WNT/b-catenin-responding mdDA domain (Figure 1C). LEF1 is a b-catenin-dependent activator of WNT target genes (Mao and Byers, 2011). Both *Lef1* mRNA and LEF1 protein were expressed prominently in a bilateral domain sparing the *Aldh1a1*^+^, *Lmx1a*^+^, and SOX6^+^ mFP but encompassing the *Wnt1*^+^ lFP and the adjacent OTX2^+^ basal plate (BP) in the E10.5 and E12.5 mouse VM (Figures 3A,E,F,J,O–S’). Notably, LEF1 expression was mostly restricted to the WNT/b-catenin-responding (b-GAL^+^) cells in the VZ/SVZ of the E12.5 *BAT-gal^+^* VM, and downregulated at the transition from the PITX3^+^ but TH^–^ IZ (containing the mdDA precursors) to the TH^+^/PITX3^+^ MZ (harboring the mdDA neurons; Figures 3T–U). At E17.5, *Lef1* mRNA was detected exclusively in the DM (superior colliculi; Figure 3K). *Tcf7l1* is a b-catenin-independent repressor of WNT target genes (Mao and Byers, 2011), and exhibited a widespread and weak expression in the entire VM at E10.5 (Figure 3C). From E12.5 onward, its transcription became restricted to the mesencephalic VZ/SVZ around the 3rd ventricle (aqueduct; Figures 3H,M). *Tcf7l2* is a context-dependent activator or repressor of WNT target genes (Mao and Byers, 2011). Its expression in the mouse midbrain initiated around E12.5 and was confined to the dorsolateral midbrain (ABB and alar plate; Figures 3D,I,N). The fourth *Lef1*/*Tcf* transcript, *Tcf7* (*Tcf1*), was not detected in neural tissues of the developing mouse midbrain (Figures 3B,G,L). These analyses confirmed the microarray data (Figure 1C) and revealed that *Lef1* and *Tcf7l1* are the only LEF1/TCF members transcribed in the murine VM at the relevant stages for mdDA neurogenesis. Moreover, they indicated that LEF1 expression is confined to an mdDA progenitor subset in the lFP, which also appears to be the WNT/b-catenin-responding (b-GAL^+^) subset in the *BAT-gal^+^* VM (compare Figures 3P–Q’, 1K–K”).

We then conducted western blot analyses of E12.5 mouse VM and DM tissues with two different LEF1 antibodies, one directed against the N- (C12A5) and the other against the C-terminus (C18A7) of this protein. This allowed us to establish whether only the activating LEF1 isoform containing the N-terminal b-catenin binding domain or also shorter, dominant-negative LEF1 isoforms due to alternative promoter usage (Mao and Byers, 2011), were expressed in the developing mouse VM. A strong band was detected in DM tissues and a much weaker band in VM tissues at the expected size (approx. 55 kDa) of full-length LEF1 protein containing the N-terminal b-catenin binding domain (Figure 3V). We concluded that the confined expression of the activating LEF1 isoform in the VZ/SVZ of the lFP restricts the response to the WNT1 ligand and other soluble agonists (e.g., RSPO2) to this particular mdDA progenitor subset in the mouse VM.

### Ectopic Expression of *Lef1* and *Lmx1a* Precedes the Ectopic Generation of *Nurr1*^+^ mdDA Precursors in the *En1*^+/*Wnt1*^ Rostral Hindbrain

**FIGURE 4:**
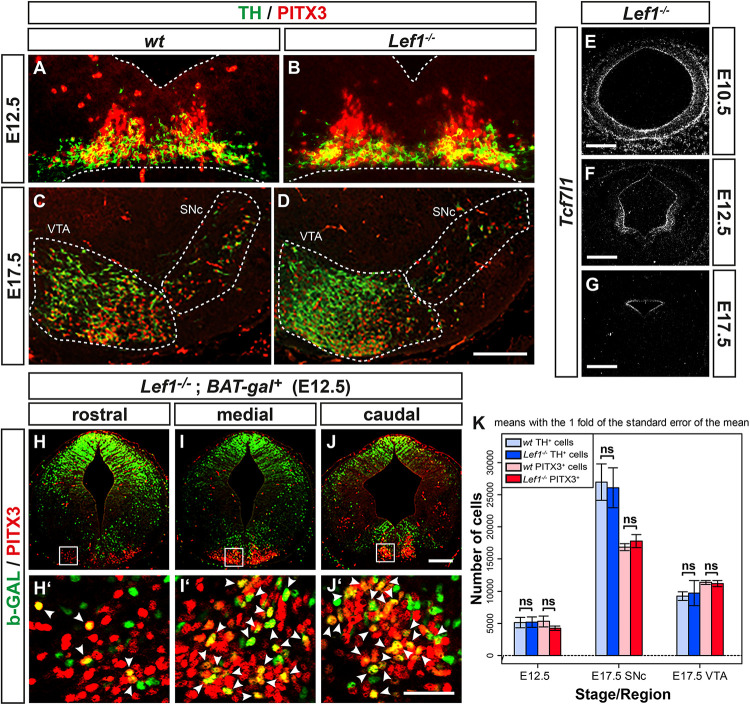
mdDA neuron numbers and WNT/b-catenin-responsiveness are not affected in the *Lef1*^–/–^ midbrain. **(A-J’)** Representative overviews **(E–J)** and close-up views of the VM (**A–D,H’–J’**, corresponding to the boxed areas in **H–J**) on coronal sections (dorsal top) at medial **(A–G)** or different rostrocaudal **(H–J’)** levels of the midbrain from *wt* (*Lef1*^+/+^ and *Lef1*^+ /−^; **A, C**) and *Lef1*^–/–^ **(B,D,E–G)** embryos at E10.5 **(E)**, E12.5 **(A,B,F),** and E17.5 **(C,D,G)**, or from *Lef1*^–/–^; *BAT-gal^+^* embryos at E12.5 **(H–J’)**. Sections were immunostained with antibodies against TH (green) and PITX3 (red; **A–D**), or b-GAL (green) and PITX3 (red; **H–J’**), overlapping expression domains appear in yellow; or hybridized with a riboprobe for *Tcf7l1* **(E–G)**. **(E–G)**, dark-field images. Broken white lines in **(A,B)** outline the neuroepithelium, and in **(C,D)** delimit the SNc and VTA. White arrowheads in **(H’–J’)** point at b-GAL^+^/PITX3^+^ double-positive cells. **(K)** Unbiased stereological quantification of TH and PITX3 single-positive cells in the E12.5 VM or E17.5 SNc and VTA of *wt* and *Lef1*^–/–^ embryos [*n* = 4 embryos/genotype/stage; ns, not significant in the Welsh *t*-test for unequal variances]. Abbreviations: SNc, Substantia nigra pars compacta; VTA, ventral tegmental area. Scale bars: 100 μm **(D)**; 200 μm **(E,F,J)**; 500 μm **(G)**; and 50 μm **(J’)**.

**FIGURE 5:**
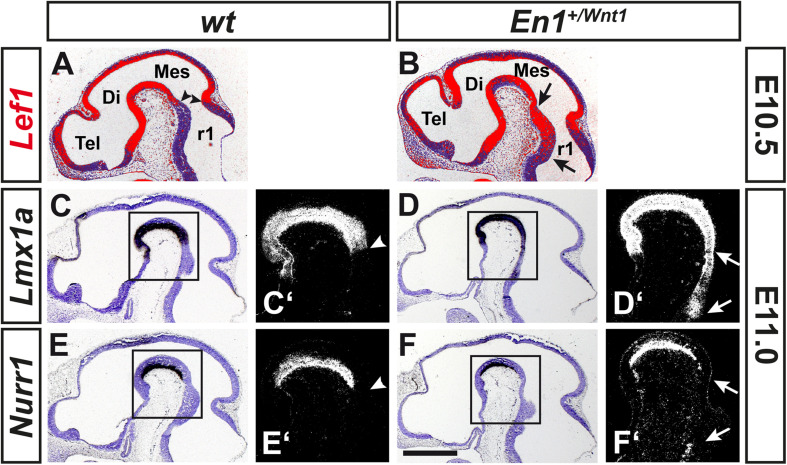
Ectopic expression of *Lef1* and *Lmx1a* precedes the ectopic generation of *Nurr1*^+^ mdDA precursors in the *En1*^+/*Wnt1*^ rostral hindbrain. **(A–F’)** Representative overviews **(A–F)** and close-up views of the cephalic flexure **(C’–F’)** on cresyl violet-stained midsagittal sections (anterior left) from *wt* (*En1*^+/+^; **A,C,C’,E,E’**) and *En1*^+/*Wnt1*^ **(B,D,D’,F,F’)** embryos at E10.5 **(A,B)** and E11.0 **(C–F’)**, hybridized with riboprobes for *Lef1* (red pseudocolor; **A,B**), *Lmx1a* **(C–D’)**, or *Nurr1* **(E–F’)**. **(C’–F’)**, higher magnification dark-field views of the boxed areas in **C–F**, respectively. Arrowheads in **(A,C’,E’)** point at the position of the mid-/hindbrain boundary, arrows in **(B,D’,F’)** delimit the region of ectopic gene induction. Abbreviations: Di, diencephalon; Mes, mesencephalon; r1, rhombomere 1; and Tel, telencephalon. Scale bar: 500 μm **(F)**.

The tight correlation between LEF1 expression in the lFP and the emergence of *Aldh1a1*^+^ mdDA progenitors and *Pitx3*^+^ mdDA precursors in this region of the murine VM, suggested that *Lef1* is involved in the generation of mdDA neurons. Therefore, we assessed a possible mdDA phenotype in *Lef1*^–/–^ mutants (van Genderen et al., 1994), and the temporal sequence of *Lef1* expression and induction of ectopic mdDA progenitors and precursors in the *En1*^+/*Wnt1*^ mouse mutant (Panhuysen et al., 2004). Unexpectedly, *Lef1*^–/–^ mice exhibited similar numbers of TH^+^ or PITX3^+^ mdDA neurons as wild-type mice (Figures 4A–D,K and Table 1). A possible reason might be the functional redundancy of LEF1 and other TCFs expressed in the murine VM. Indeed, transcription of *Tcf7l1* was unaffected in the *Lef1*^–/–^ midbrain (Figures 4E–G). Moreover, b-GAL expression in the *Lef1*^–/–^; *BAT-gal^+^* midbrain at E12.5 was similar to *wt BAT-gal^+^* mice (compare Figures 4H–J with Supplementary Figures 5A–C), although the proportion of WNT/b-catenin-responding (b-GAL^+^) and PITX3^+^ mdDA precursors and neurons appeared to be slightly increased in the mediocaudal VM of the *Lef1*^–/–^ mutant embryos (compare Figures 4H’–J’ with Supplementary Figures 5A’–C’). In *En1*^+/*Wnt1*^ mice, *Wnt1* expression is ectopically expanded into the rostral hindbrain and ectopic mdDA neurons are generated in the FP of the mutant rostral hindbrain (Panhuysen et al., 2004; Prakash et al., 2006). In the E10.5–11.5 *wt* mouse embryo, the expression of *Lef1* exhibited a clear caudal border at the mid-/hindbrain boundary and was not detected in the rostral hindbrain (rhombomere 1, r1; Figure 5A; Oosterwegel et al., 1993). In the E10.5–11.5 *En1*^+/*Wnt1*^ embryo, however, the posterior *Lef1* expression border had shifted caudally into the r1 FP (Figure 5B; Panhuysen et al., 2004). This ectopic (posterior) expansion of *Lef1* expression correlated with the ectopic induction of the mdDA progenitor marker *Lmx1a* in the FP of the *En1*^+/*Wnt1*^ rostral hindbrain at E11.0 (Figures 5C–D’). Notably, the postmitotic mdDA precursor marker *Nurr1* (*Nr4a2*) was not detected in the FP of the *En1*^+/*Wnt1*^ rostral hindbrain before E12.5 (Figures 5E–F’; Prakash et al., 2006). We concluded that ectopic *Wnt1* and *Lef1* are sufficient for ectopic induction of *Lmx1a*^+^ mdDA progenitors in the FP of the *En1*^+/*Wnt1*^ rostral hindbrain. The emergence of postmitotic *Nurr1*^+^ mdDA precursors, however, was delayed in relation to the ectopic expression of *Lef1* in this region of the *En1*^+/*Wnt1*^ mutant hindbrain. Altogether, our data indicated that *Lef1* is dispensable for the generation of mature mdDA neurons or its lack is compensated by another LEF1/TCF TF, most likely *Tcf7l1*, in the *Lef1*^–/–^ VM. LEF1, however, appears to be causally related to ectopic WNT1-mediated signaling and the ectopic induction of mdDA progenitors in the *En1*^+/*Wnt1*^ mutant mouse model.

**TABLE 1:** Unbiased stereological counts of TH^+^ and PITX3^+^ single-positive cells in the VM of *wt* and *Lef1*^–/–^ embryos.

Age	Region	Genotype	TH^+^ cells (Mean ± s.e.m.)	PITX3^+^ cells (Mean ± s.e.m.)	*n*
E12.5	VM	*wt*	5127 ± 807	5316 ± 832	4
		*Lef1*^–/–^	5180 ± 797	4240 ± 345	4
E17.5	SNc	*wt*	26924 ± 2822	16837 ± 529	4
		*Lef1*^–/–^	26071 ± 3071	17771 ± 1013	4
	VTA	*wt*	9255 ± 660	11362 ± 300	4
		*Lef1*^–/–^	9725 ± 1957	11194 ± 461	4

*SNc, substantia nigra pars compacta; VM, ventral midbrain; and VTA, ventral tegmental area.*

### The Murine *Pitx3* Promoter Is Dose-Dependently Repressed by LEF1-Mediated WNT/b-Catenin Signaling

**FIGURE 6:**
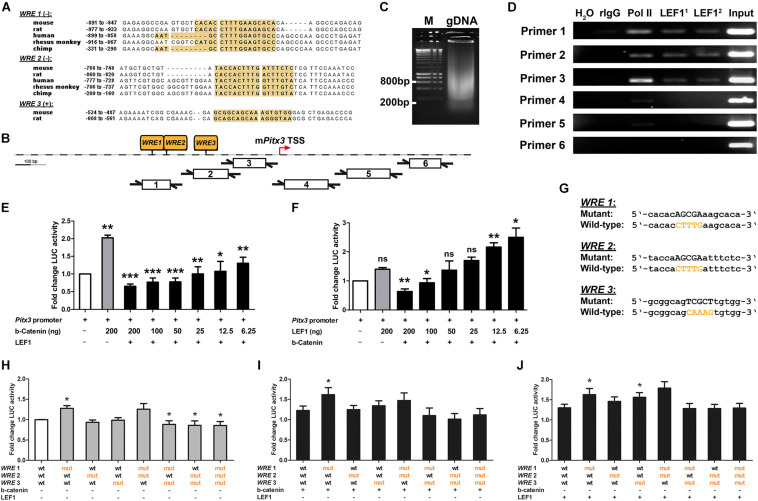
The murine *Pitx3* promoter is dose-dependently repressed by LEF1-mediated WNT/b-catenin signaling. **(A)** Alignments of conserved LEF1/TCF BSs/*WREs* (yellow boxes) in *Pitx3* promoter regions of mouse, rat, human, rhesus monkey, and chimp (*WRE 1*/*2*, minus strand), or mouse and rat (*WRE 3*, plus strand). **(B)** Position of the three conserved LEF1/TCF BSs/*WREs* (*WRE 1-3*) in the mouse *Pitx3* (m*Pitx3*) gene promoter relative to the transcription start site (TSS), and of the six primer pairs used in ChIP-PCR analysis. **(C)** Sheared genomic DNA (gDNA) isolated from the VM of E11.5 *CD-1* embryos. M, DNA ladder. **(D)** Representative ChIP-PCR analysis of LEF1 binding to the m*Pitx3* promoter in the E11.5 mouse VM using six overlapping primer pairs (Primer 1-6). H_2_O, negative PCR control; rIgG, negative ChIP control; Pol II, positive ChIP control; LEF1^1^, LEF1 mAb C12A5; LEF1^2^, LEF1 mAb C18A7; and input, sheared E11.5 mouse VM gDNA. **(E,F)** Fold change of luciferase (LUC) activity in HEK-293 cells (relative to *Pitx3* promoter/reporter only-transfected cells, set as 1) after transfection of decreasing amounts of *S33Y-b-catenin* or *LEF1* cDNA alone or together with *LEF1* or *S33Y-b-catenin* plasmids, respectively, [*n* = 3 (*LEF1* gradient) and 4 (*b-catenin* gradient) independent experiments; statistical testing for significance was done between m*Pitx3* promoter/reporter only-transfected cells (white bars) and *Pitx3* promoter + *S33Y-b-catenin* or *LEF1*-transfected cells (gray bars), and between the latter and cells transfected with decreasing amounts of *S33Y-b-catenin* or *LEF1* plasmids in the presence of *LEF1* or *S33Y-b-catenin* (black bars), respectively, using one-way ANOVA followed by Bonferroni’s multiple comparisons *post hoc* tests; F(7,23/16) = 8.0/12.0, *P* < 0.0001]. **(G)** Alignment of mutant LEF1/TCF BSs/*WREs* (*WRE 1-3*) and *wt* counterparts in the m*Pitx3* promoter. **(H–J)** Fold change of luciferase (LUC) activity in HEK-293 cells [relative to *wt Pitx3* promoter/reporter only-transfected cells, set as 1; white bar in **(H)**] after transfection of mutant *WRE 1*-*3 Pitx3* promoter/reporter alone [gray bars in **(H)**, *n* = 4 independent experiments] or together with *S33Y-b-catenin* **(I)** or *LEF1* cDNA **(J)** [*n* = 4 (+*S33Y-b-catenin*) and 7 (+*LEF1*) independent experiments; statistical testing for significance was done between *wt* and mutant *WRE 1-3 Pitx3* promoter/reporter-transfected cells in each condition using Wilcoxon–Mann–Whitney test]. *P < 0.05; **P < 0.005; ***P < 0.0001; and ns, not significant.

Our results so far indicated that “canonical” WNT/b-catenin signaling is activated by WNT1 and RSPO2 on the cell surface and mediated by nuclear LEF1 in an LMX1A^+^ mdDA progenitor subset located in the VZ/SVZ of the mesencephalic lFP. This WNT1/b-catenin signal presumably has to be downregulated in LMX1A^+^ mdDA precursors before their differentiation into PITX3-expressing mdDA neurons. Whereas *Lmx1a* is an established direct target gene of WNT1- and LEF1-mediated signaling in the mouse VM (Chung et al., 2009; Zhang et al., 2015), such evidence is lacking for *Pitx3*. We thus determined whether *Pitx3* is a direct target gene of this signaling pathway. Three conserved (by sequence similarity and position) LEF1/TCF BSs containing the canonical *WRE* sequence [5′-[A/T][A/T]CAAAG-3′ in sense, 5′-CTTTG[T/A][T/A]-3′ in antisense orientation (Haynes et al., 1996)] were predicted upstream of the TSS in the *Pitx3* promoter regions of several mammalian species, including mouse, rat and human (Figures 6A,B). ChIP of genomic DNA isolated from the E11.5 mouse VM showed that these LEF1/TCF BSs/*WREs* in the native *Pitx3* promoter were indeed bound by endogenous LEF1 protein (Figures 6C,D). The native *Pitx3* promoter region was also bound by RNA polymerase II (Pol II), indicating that this gene was being actively transcribed in the E11.5 mouse VM (Figure 6D).

We then determined the effect of WNT/b-catenin signaling on transcription from a previously characterized ∼2.5 kb *Pitx3* promoter fragment containing the three conserved LEF1/TCF BSs/*WREs* (Peng et al., 2011). We performed these experiments in heterologous HEK-293 cells, a widely used WNT/b-catenin-responding (BAT-gal^+^) cell line expressing detectable amounts of *LEF1* mRNA and nuclear LMX1A protein (Supplementary Figure 2; Alok et al., 2017). As expected, transfection of constitutively active (stabilized) *S33Y-b-catenin* into HEK-293 cells resulted in the strong activation of a *TOPFlash* luciferase reporter containing seven multimerized LEF1/TCF BSs/*WREs* (Supplementary Figure 2). Decreasing amounts of *S33Y-b-catenin* in the presence of co-transfected full-length human *LEF1* led to a significant reduction of luciferase activity (Supplementary Figure 2). Transfection of *LEF1* alone had no effect on *TOPFlash* reporter activity, and co-transfection of decreasing amounts of *LEF1* together with *S33Y-b-catenin* led to a variable but not significant activation of the *TOPFlash* reporter (Supplementary Figure 2). These results indicated that in WNT/b-catenin-responding HEK-293 cells, the activation of an artificial WNT target gene/reporter construct is limited by the amounts of stabilized (unphosphorylated) b-catenin but not LEF1 in these cells. As expected from the previous results, transfection of constitutively active (stabilized) *S33Y-b-catenin* alone led to a 2 ± 0.075-fold activation of the mouse *Pitx3* promoter, whereas transfection of *LEF1* alone had no significant effect on this promoter in HEK-293 cells (Figures 6E,F). Surprisingly, co-transfection of decreasing amounts of stabilized *S33Y-b-catenin* in the presence of *LEF1* resulted in a 0.65 ± 0.065 to 0.78 ± 0.11-fold repression of the *Pitx3* promoter/reporter construct at high *S33Y-b-catenin* levels. A 1.1 ± 0.28 to 1.3 ± 0.17-fold activation of this promoter was only detected with the two lowest amounts (12.5 and 6.25 ng) of *S33Y-b-catenin* tested in this context (Figure 6E). Consistent with this result, only the two lowest *LEF1* doses (12.5 and 6.25 ng) caused a 2.2 ± 0.15 to 2.5 ± 0.32-fold activation of the *Pitx3* promoter/reporter construct, whereas high *LEF1* amounts (200 and 100 ng) led to a 0.63 ± 0.088 to 0.93 ± 0.15-fold repression of this promoter after co-transfection of *S33Y-b-catenin* (Figure 6F). Together, these data indicated that the mouse *Pitx3* promoter is repressed rather than activated by high levels of WNT/b-catenin/LEF1-mediated signaling (i.e., in the presence of high doses of stabilized b-catenin and LEF1). This repression is turned into an activation of the *Pitx3* promoter only when the levels of stabilized b-catenin or LEF1 protein are strongly decreased in the responding cell.

Next, we determined whether the repressive effect of strong WNT/b-catenin/LEF1 signaling on the murine *Pitx3* promoter is in fact mediated by the three conserved, LEF1-bound BSs/*WREs* in this promoter, through mutagenizing the *WRE* core sequence [5′-CAAAG-3′ or 5′-CTTTG-3′] in each of these three LEF1/TCF BSs (Figure 6G). Mutation of *WRE 1* in the most distal (relative to the *Pitx3* TSS) LEF1/TCF BS alone consistently resulted in a significant (*P* = 0.012, *P* = 0.013, and *P* = 0.033, respectively) 1.27 ± 0.06 to 1.62 ± 0.15-fold de-repression (activation) of the mouse *Pitx3* promoter, both in the complete absence and presence of high amounts (200 ng) of co-transfected *S33Y-b-catenin* or *LEF1* (Figures 6H–J). Mutation of *WRE 3* in the most proximal (relative to the *Pitx3* TSS) LEF1/TCF BS also resulted in a significant (*P* = 0.016) 1.56 ± 0.11-fold de-repression (activation) of the mouse *Pitx3* promoter in the presence of high amounts of *LEF1* (Figure 6J), indicating that LEF1-binding to *WRE 1* and *3* represses the murine *Pitx3* promoter. Mutation of *WRE 3* together with *WRE 1* and/or *WRE 2* (in the intermediate LEF1/TCF BS) led to a small but significant (*P* = 0.039) 0.85 ± 0.09 to 0.88 ± 0.09-fold reduction of luciferase activity in the absence of co-transfected *S33Y-b-catenin* and *LEF1* (Figure 6H), and a similar but non-significant trend in the presence of *S33Y-b-catenin* or *LEF1* (Figures 6I,J). This result suggested that *WRE 3* together with *WRE 1* and/or *WRE 2* directly or indirectly (via other TFs binding in the same region) contribute to the activation of the mouse *Pitx3* promoter.

**FIGURE 7:**
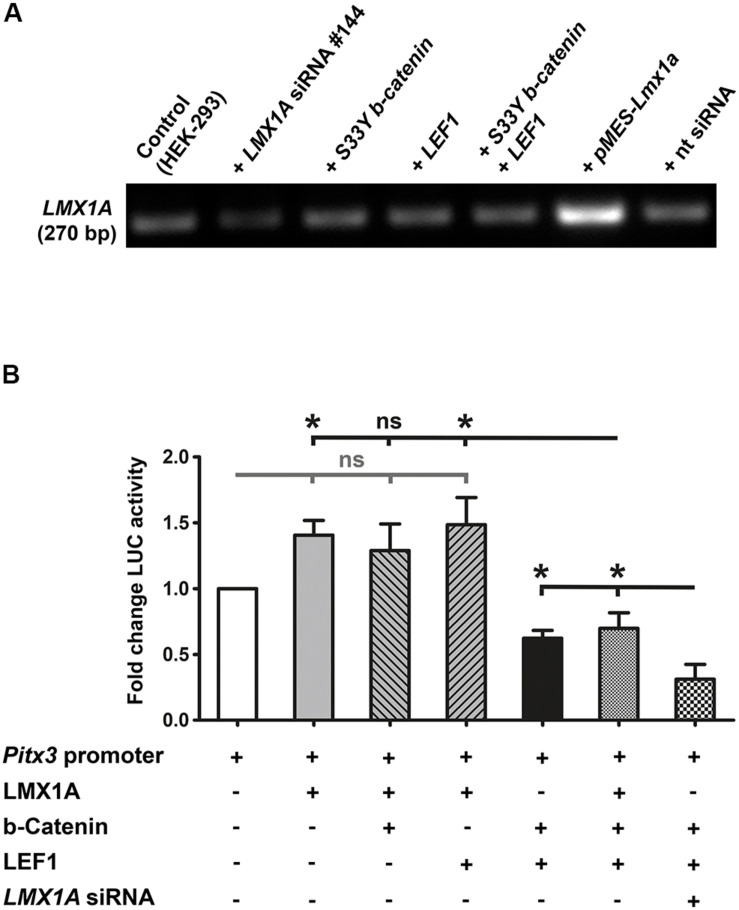
LMX1A-mediated activation of the murine *Pitx3* promoter is suppressed in the presence of high LEF1/b-catenin levels. **(A)** RT-PCR analyses of total RNA isolated from untransfected HEK-293 cells (control), or after transfection of *LMX1A* siRNA (#144), 200 ng/well constitutively active *S33Y-b-catenin* and *LEF1* cDNA alone or together, *pMES-Lmx1a* (encoding the full-length mouse LMX1A protein) or non-targeting (*nt*) siRNA (control), for the detection of *LMX1A* transcription in these cells. Densitometric analyses (normalized to *Gapdh* mRNA levels, not shown) revealed an approx. 50% reduction of endogenous *LMX1A* mRNA levels in *LMX1A* siRNA-transfected HEK-293 cells, and an approx. 300% increase of *LMX1A* transcripts in these cells after transfection of *pMES-Lmx1a*. All other conditions had no detectable influence on endogenous *LMX1A* transcript levels in HEK-293 cells. **(B)** Fold change of luciferase (LUC) activity in HEK-293 cells (relative to *Pitx3* promoter/reporter only-transfected cells, set as 1; white bar) after co-transfection with 166 ng/well *Lmx1a*, 200 ng/well *S33Y-b-catenin*, 200 ng/well *LEF1*, and *LMX1A* siRNA (#144) alone or together in different constellations, as indicated below the graph [*n* = 3 independent experiments; statistical testing for significance was done using one-way ANOVA followed by Bonferroni’s multiple comparisons *post hoc* tests; F(6,14) = 10.92, *P* < 0.0001]. *P < 0.05; ns, not significant.

Because LMX1A is a direct target gene of WNT1/b-catenin/LEF1 signaling in the mouse VM and binds directly to the murine *Pitx3* promoter region *in vivo* (Chung et al., 2009), we also determined whether LMX1A could mediate the activating effects of this signaling pathway on the *Pitx3* promoter *in vitro*. Co-transfection of the mouse *Pitx3* promoter/reporter construct and a plasmid encoding the full-length mouse LMX1A protein [*pMES-Lmx1a*, (Klafke et al., 2016)] consistently resulted in a 1.5- to 2-fold (in some cases significant, data not shown) activation of this promoter (Figures 7A,B). Co-transfection of stabilized *S33Y-b-catenin* or *LEF1* together with *Lmx1a*, however, did not result in a further increase of LMX1A-mediated activation of the *Pitx3* promoter/reporter construct (Figure 7B). This was mirrored by apparently unaltered *LMX1A* mRNA levels in HEK-293 cells transfected with the stabilized *S33Y-b-catenin* and/or *LEF1* plasmids (Figure 7A). Notably, co-transfection of all three plasmids (*Lmx1a*, *S33Y-b-catenin* and *LEF1*) together resulted in a significant decrease of *Pitx3* promoter activity relative to *Lmx1a* alone (*P* = 0.0123) or *Lmx1a* and *LEF1* co-transfected (*P* = 0.0297) cells (Figure 7B). Repression of the *Pitx3* promoter under this condition was similar to the repressive effect on this promoter of high *S33Y-b-catenin* and *LEF1* levels alone (Figure 7B). We also performed *LMX1A* siRNA knock-down experiments to lower the endogenous LMX1A content in HEK-293 cells (Figure 7A). Reduction of endogenous *LMX1A* mRNA levels to approx. 50% resulted in an even more pronounced and significant (*P* = 0.0370 relative to *S33Y-b-catenin* + *LEF1; P* = 0.0172 relative to *Lmx1a* + *S33Y-b-catenin* + *LEF1*) ∼70% repression of the *Pitx3* promoter in the presence of high amounts of *S33Y-b-catenin* and *LEF1* (Figure 7B). These results strongly indicated that the activation of the mouse *Pitx3* promoter by LMX1A is suppressed in the presence of high WNT1/b-catenin/LEF1 signaling levels in HEK-293 cells. Altogether, our data revealed that the murine *Pitx3* promoter is a direct target of WNT/b-catenin/LEF1-mediated signaling and repressed by a high dosage of this signaling pathway, which also inhibits its activation by LMX1A.

### WNT/b-Catenin-Responding mdDA Progenitors Are Restricted Mostly to the Lateral FP of the Medial and Caudal Mouse Midbrain

Based on the previous results, we determined whether the restricted expression of LEF1 in the mouse VM, particularly in the lFP (Figure 3), correlated with the WNT/b-catenin-responsiveness of these cells. Firstly, we compared the b-GAL expression in two different but established reporter mouse strains for WNT/b-catenin signaling, *TOPGAL* (DasGupta and Fuchs, 1999), and *BAT-gal* (Maretto et al., 2003), to the LEF1 expression pattern determined before (Figure 3). Only very few b-GAL^+^ (i.e., WNT/b-catenin-responding) cells were detected in the DM (roof plate) of the E12.5 *TOPGAL*^+^ mouse embryo, whereas the remaining midbrain tissue was b-GAL^–^, which means non-WNT/b-catenin-responding (Supplementary Figure 3). *BAT-gal* mouse embryos, by contrast, reproducibly displayed a b-GAL expression pattern that was very similar to LEF1 expression in the mouse midbrain (Figures 1A,K–K”,8A,E,I,M, and Supplementary Figures 5A–C). We concluded that the *TOPGAL* mouse, in which b-GAL is expressed upon activation of a minimal promoter containing three consensus LEF1/TCF BSs, is not a good reporter for ongoing WNT/b-catenin signaling in the mouse midbrain.

**FIGURE 8:**
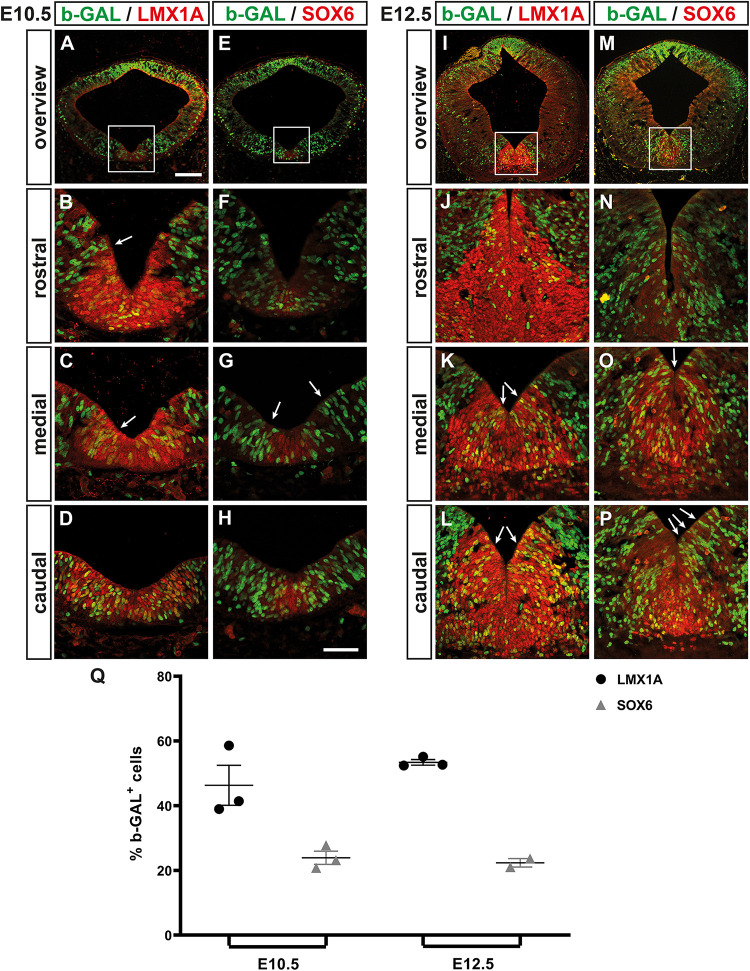
WNT/b-catenin-responding mdDA progenitors are restricted mostly to the lFP of the medial and caudal mouse midbrain. **(A–P)** Representative overviews **(A,E,I,M)** and close-up views of the VM (**B–D,F–H,J–L,N–P;C,G,K,O** correspond to boxed areas in **A,E,I,M**, respectively) on coronal sections (dorsal top) at different rostrocaudal levels of the *BAT-gal^+^* midbrain at E10.5 **(A–H)** and E12.5 **(I–P)**, immunostained with antibodies against b-GAL (green) and LMX1A (red; **A–D,I–L**) or SOX6 (red; **E–H,M–P**); overlapping expression domains appear in yellow. White arrows in **(B,C,G,K,L,O,P)** point at “chains” of b-GAL^+^ cells. **(Q)** Quantification of the proportion of b-GAL^+^/LMX1A^+^ and b-GAL^+^/SOX6^+^ double-positive cells among all LMX1A^+^ or SOX6^+^ cells, respectively, in the VM of E10.5 and E12.5 *BAT-gal^+^* embryos [*n* = 3 embryos/stage; E10.5, b-GAL^+^/LMX1A^+^: 46.33 ± 6.17%; b-GAL^+^/SOX6^+^: 23.90 ± 2.04%; E12.5, b-GAL^+^/LMX1A^+^: 53.41 ± 0.87%; b-GAL^+^/SOX6^+^: 22.35 ± 1.30%; and *P* = 0.0183 in Kruskal–Wallis with Dunn’s multiple comparisons *post hoc* test]. Scale bars: 100 μm **(A)**; 50 μm **(H)**.

We then determined the location and overall proportion of WNT/b-catenin-responding (b-GAL^+^) mdDA progenitors in the midgestational *BAT-gal* mouse embryo. At the beginning of mdDA neurogenesis [E10.5; (Bye et al., 2012)], 46.33 ± 6.17% of the LMX1A^+^ FP (mdDA) progenitors were also b-GAL^+^ and preferentially located in the medial and caudal VM (Figures 8A–D,Q). Only 23.90 ± 2.04% of the SOX6^+^ mFP cells (Panman et al., 2014) co-expressed b-GAL at this stage (Figures 8E–H,Q). Thus, WNT/b-catenin-responding cells were mostly detected within the LMX1A^+^/SOX6^–^ lFP and the adjacent LMX1A^–^ BP of the E10.5 VM (Figures 8A–H). This pattern strongly resembled *Lef1*/LEF1 expression in the *wt* mouse VM at this stage (Figure 3). At the peak of mdDA neurogenesis [E12.5; (Bye et al., 2012)], 53.41 ± 0.87% of the LMX1A^+^ FP cells but only 22.35 ± 1.30% of the SOX6^+^ mFP cells were WNT/b-catenin-responding (b-GAL^+^; Figures 8I–Q). The b-GAL^+^/LMX1A^+^ double-positive cells still spared the mFP and were mostly detected in the mediocaudal *BAT-gal* VM (Figures 8I–L). This again correlated strongly with the *Lef1*/LEF1 expression pattern in the *wt* VM at E12.5 (Figure 3). Notably, we frequently observed “chains” of b-GAL^+^ cells extending from the VZ/SVZ to the MZ within and outside the mdDA domain at E10.5 and E12.5 (Figures 8B,C,G,K,L,O,P). This suggested a clonal relationship between these cells, which might represent migrating precursors and/or neurons that have already ceased to respond to a WNT/b-catenin signal. Together, our data revealed a good correlation between LEF1 expression in the midgestational mouse VM and the WNT-b-catenin-responsiveness of the corresponding cells. This population comprised only half of the LMX1A^+^ mdDA progenitors/precursors and was located preferentially in the SOX6^–^ lFP of the medial and caudal mouse VM. We concluded that only a subset of the mdDA progenitors, most likely co-expressing all necessary WNT/b-catenin signaling pathway components (including *Wnt1*, *Rspo2*, and *Lef1*), is responding to this pathway in the midgestational mouse VM.

### WNT/b-Catenin-Responding Cells Give Rise to Only a Fraction of all mdDA Precursors and Neurons Throughout Development

**FIGURE 9:**
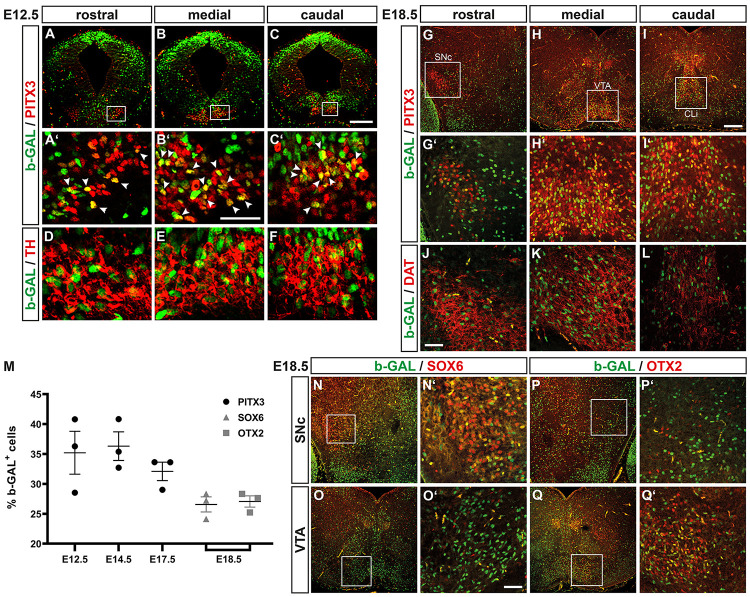
WNT/b-catenin-responding cells give rise to only a fraction of all mdDA precursors and neurons throughout development. **(A–L,N–Q’)** Representative overviews **(A–C,G–I,N–Q)** and close-up views of the VM (**A’–F,G’–L,N’–Q’; A’–C’,G’–I’**; **N’–Q’** correspond to boxed areas in **A–C,G–I,N–Q**, respectively) on coronal sections (dorsal top) at different rostrocaudal levels of the *BAT-gal^+^* midbrain at E12.5 **(A–F)** and E18.5 **(G–L,N–Q’)**, immunostained with antibodies against b-GAL (green) and PITX3 (red; **A–C’,G–I’**), TH (red; **D–F**), DAT (red; **J–L**), SOX6 (red; **N–O’**), or OTX2 (red; **P–Q’**); overlapping expression domains appear in yellow. White arrowheads in **(A’–C’)** point at b-GAL^+^/PITX3^+^ double-positive cells. **(M)** Quantification of the proportion of b-GAL^+^/PITX3^+^, b-GAL^+^/SOX6^+^, and b-GAL^+^/OTX2^+^ double-positive cells among all PITX3^+^, SOX6^+^, or OTX2^+^ cells, respectively, in the VM of E12.5, E14.5, and E17.5 or E18.5 *BAT-gal^+^* embryos [*n* = 3 embryos/stage; b-GAL^+^/PITX3^+^: E12.5, 35.21 ± 3.59%; E14.5, 36.31 ± 2.39%; E17.5, 32.09 ± 1.54%; b-GAL^+^/SOX6^+^: E18.5, 26.57 ± 1.26%; b-GAL^+^/OTX2^+^: E18.5, 27.06 ± 0.94%; and *P* = 0.0063 in Kruskal–Wallis with Dunn’s multiple comparisons *post hoc* test]. Abbreviations: CLi, caudal linear nucleus of the raphe; SNc, Substantia nigra pars compacta; and VTA, ventral tegmental area. Scale bars: 200 μm **(C,I)**; 100 μm **(O’)**; and 50 μm **(B’,J)**.

The b-GAL protein is known to be very stable (Jacobsen and Willumsen, 1995) and might thus persist in the nucleus even though WNT/b-catenin signaling has already ceased to act in this cell. Indeed, b-GAL^+^ cells were detected throughout the FP neuroepithelium (including the LEF1^–^ but TH^+^ MZ) in the E12.5 *BAT-gal^+^* VM (Figures 3T–T”, 8I–P), suggesting that the b-GAL protein persisted in maturing TH^+^ mdDA neurons even though these cells were not capable of responding to a WNT/b-catenin signal anymore. Taking this into consideration, we determined the proportion of b-GAL^+^ and PITX3^+^ cells in the *BAT-gal^+^* VM. At E12.5, 35.21 ± 3.59% of the PITX3^+^ cells co-expressed b-GAL (Figures 9A–C’,M). The b-GAL^+^/PITX3^+^ and b-GAL^+^/TH^+^ mdDA precursors and neurons were mostly located in the mediocaudal *BAT-gal^+^* midbrain, whereas fewer double-positive cells were detected in the rostral midbrain (Figures 9A–F). At E14.5 and E17.5, 36.31 ± 2.39% and 32.09 ± 1.54%, respectively, of the PITX3^+^ cells were also b-GAL^+^ (Figure 9M). These b-GAL^+^ cells co-expressed DAT, a marker for mature mdDA neurons, and were mostly detected in the medial midbrain (VTA) and in the caudal linear nucleus of the raphe (CLi) at E18.5 (Figures 9G–L).

Because the mediocaudal lFP, which is hypothesized to give preferentially rise to VTA DA neurons (Bodea and Blaess, 2015), was prominently LEF1^+^ and b-GAL^+^ (WNT/b-catenin-responding) in the midgestational *BAT-gal^+^* VM (Figures 3, 8), we determined the co-expression of b-GAL in SOX6^+^ SNc DA neurons (Figures 9N–O’; Panman et al., 2014) and OTX2^+^ VTA DA neurons (Figures 9P–Q’; Di Salvio et al., 2010; Panman et al., 2014) at E18.5. A similar proportion of SOX6^+^ SNc DA (26.57 ± 1.26%) and OTX2^+^ VTA DA (27.06 ± 0.94%) neurons were also b-GAL^+^ at this stage (Figure 9M). Notably, b-GAL expression was strongly downregulated in the SNc, VTA and CLi of adult (3 months-old) *BAT-gal^+^* mice (Supplementary Figure 4), supporting the notion that the b-GAL-positivity of postmitotic mdDA neurons is only transient and due to the stability of the b-GAL protein in the cells’ nucleus.

Lastly, we determined whether activation of the WNT/b-catenin pathway in the mouse FP is achieved exclusively by WNT1 or also by other WNT ligands. Heterozygote *Wnt1*^+ /−^; *BAT-gal^+^* embryos showed a similar b-GAL expression pattern and proportion of b-GAL^+^/PITX3^+^ cells as *Wnt1*^+/+^; *BAT-gal^+^* embryos at E12.5 (Figures 9A–C’ and Supplementary Figures 5A–C’). However, b-GAL^+^ cells were drastically reduced in the VM of homozygote *Wnt1*^–/–^; *BAT-gal^+^* embryos at this stage (Supplementary Figure 5D). Notably, the few remaining and laterally displaced PITX3^+^ and TH^+^ mdDA neurons in the *Wnt1*^–/–^; *BAT-gal^+^* VM were invariably b-GAL^–^, i.e., non-WNT/b-catenin-responding (Supplementary Figures 5D’,E). Altogether, these data suggested that approximately one third of the postmitotic mdDA precursors are derived from WNT1/b-catenin-responding mdDA progenitors, and contribute equally to SNc and VTA DA neurons despite their preferential location in the lFP of the mediocaudal mouse midbrain. Moreover, WNT1 appears to be the main activating WNT ligand in this context.

## Discussion

Here, we provide evidence that the WNT/b-catenin signaling pathway plays an important and subset-specific regulatory function not only in mdDA progenitor proliferation and cell fate specification, but also during their differentiation into mature mdDA neurons *in vitro* and most likely also *in vivo*.

### Dose-Dependent and Subset-Specific Inhibition of mdDA Neuron Differentiation by RSPO2- and LEF1-Mediated WNT1/b-Catenin Signaling

The microarray-based transcriptome profiling of the WNT/b-catenin-responding mdDA domain in the *BAT-gal* VM and subsequent expression pattern analysis of candidate genes revealed that the postmitotic mdDA precursor/neuron marker *Pitx3* and two activating components of the WNT/b-catenin pathway, *Rspo2* and *Lef1*, are expressed in mutually exclusive domains in the mouse VM. RSPO2 is a potent activator or enhancer of WNT/b-catenin signaling that inhibited the differentiation of E11.5 VM progenitors into maturing PITX3^+^/TH^+^ mdDA neurons in a dose-dependent manner and without affecting their proliferation [this study, (Kim et al., 2008)]. A recent report showed the opposite effect (increased dopaminergic differentiation) after treatment of E11.5 mouse VM primary cultures with the same recombinant human RSPO2 protein used in our study (Gyllborg et al., 2018). Although we can only speculate about possible reasons for this discrepancy, one notable difference between both studies was the amount and timing of RSPO2 addition. Gyllborg et al. (2018) applied a much higher RSPO2 concentration (160 ng/ml) for a shorter period of time (3 instead of 7 days), which according to our own data, do not lead to a further activation of WNT/b-catenin signaling but to a slight albeit insignificant recovery of PITX3^+^/TH^+^ mdDA neuron numbers in the treated cultures (Figures 2I,J). Notably, RSPO2 had no effect on VM progenitor proliferation in both contexts [this study, (Gyllborg et al., 2018)]. Deletion of *Rspo2* in the mouse results in a slight loss of TH^+^, PITX3^+^ and ALDH1A1^+^ cells (Hoekstra et al., 2013). This loss presumably affects mostly the rostrolateral (SNc) mdDA domain and was speculated to be due to the premature cell cycle exit and differentiation of the *Rspo2*^–/–^ mutant mdDA progenitors (Hoekstra et al., 2013). The lack of a detailed phenotypic analysis in the study by Hoekstra et al. (2013) makes it difficult to establish the precise contribution of RSPO2-mediated WNT/b-catenin signaling to the development of specific mdDA neuron subsets *in vivo*. We speculate that the *Rspo2*^–/–^ mutants, similar to the conditional *Ctnnb1* and *Wnt1* mutants, represent a WNT/b-catenin loss-of-function mouse model that might affect primarily the differentiation of mediocaudal (VTA) mdDA neurons (see model below). Moreover, the almost exclusive detection of the LGR4/5 receptors for RSPO2 in progenitor cells located in the VZ/SVZ of the mouse VM (Gyllborg et al., 2018) strongly suggests that the physiological activity of RSPO2 is rather confined to these cells.

**FIGURE 10:**
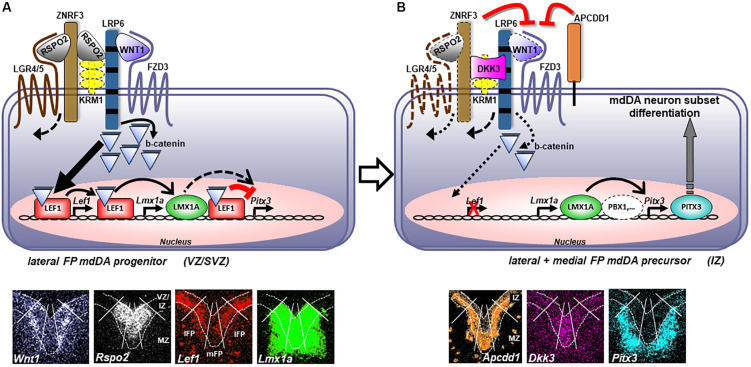
Dose-dependent regulation of mdDA neuron subset differentiation by LEF1-mediated WNT1/b-catenin signaling. **(A)** Proliferating mdDA progenitors in the VZ/SVZ of the mesencephalic lFP co-express *Wnt1*, *Rspo2*, *Lef1*, *Lmx1a*, *Fzd3* (Fischer et al., 2007; Stuebner et al., 2010), *Lrp6* (Castelo-Branco et al., 2010), *Lgr4*/*5* and *Znrf3* (Gyllborg et al., 2018), but not *Pitx3*. Binding of WNT1 to the FZD3/LRP6 receptor/co-receptor complex initiates the WNT/b-catenin signaling cascade that is enhanced by binding of RSPO2 to inhibitory KRM1 or ZNRF3/LGR4/5 complexes on the surface of these cells, thereby resulting in their internalization. Stabilized cytosolic b-catenin translocates into the nucleus of the lFP mdDA progenitors and activates *Lef1* transcription in these cells. Binding of stabilized b-catenin to LEF1, in turn, initiates or potentiates the transcription of LEF1-bound *Lmx1a* (Zhang et al., 2015) and several other genes involved in proliferation and/or cell cycle control of these WNT/b-catenin-responding lFP mdDA progenitors. At the same time, binding of stabilized b-catenin to LEF1 represses the activation of the LEF1-bound *Pitx3* promoter by LMX1A, thereby inhibiting their precocious differentiation into PITX3^+^ mdDA precursors. **(B)** Proliferating mdDA progenitors as well as postmitotic mdDA precursors in the mFP, or transiting from the VZ/SVZ to the MZ in the IZ of the mesencephalic lFP, co-express inhibitory *Dkk3* (Fukusumi et al., 2015) and/or *Apcdd1*, together with *Lmx1a*, *Fzd3* (Fischer et al., 2007; Stuebner et al., 2010), *Lrp6* (Castelo-Branco et al., 2010), and possibly *Wnt1*, *Rspo2*, *Lgr4*/*5*, and *Znrf3* (Gyllborg et al., 2018). Attenuation of a potentially still ongoing WNT1/b-catenin signal transduction by APCDD1 and DKK3, and lack of *Lef1* transcription in these cells, relieves the *Pitx3* promoter from its LEF1/b-catenin-mediated repression. The *Pitx3* promoter can now be activated by other TFs, such as LMX1A (Chung et al., 2009) and PBX1 (Villaescusa et al., 2016), expressed in postmitotic and migrating mdDA precursors. PITX3 expression in these cells promotes their differentiation into an mdDA neuron subset. Bottom panels depict single pseudocolored ISH images of the overlay shown in Figure 3O (except for *Rspo2*, *Apcdd1*, and *Dkk3*); white lines delimit lFP from mFP, broken white lines delimit VZ/IZ from MZ. Expression of *Krm1* in the murine mdDA domain has not been demonstrated yet. Abbreviations: APCDD1, adenomatosis polyposis coli down-regulated 1; FZD3, frizzled class receptor 3; KRM1, kringle containing transmembrane protein 1 (Kremen1); LGR4/5, leucine-rich repeat-containing G protein-coupled receptor 4/5; LRP6, low density lipoprotein receptor-related protein 6; and ZNRF3, zinc and ring finger 3.

*Lmx1a* is an established direct target of LEF1-mediated WNT1/b-catenin signaling during mdDA neuron generation *in vivo* (Chung et al., 2009; Zhang et al., 2015). We show now that the murine *Pitx3* gene is also a direct target of this signaling pathway in the developing mouse VM. Unexpectedly, LEF1 repressed the activation of the *Pitx3* promoter upon stabilized b-catenin stimulation in a dose-dependent manner and through direct binding of conserved *WREs* in this promoter. Based on these and recent stem cell-derived data (de Gregorio et al., 2018), we propose a mechanistic model of RSPO2- and LEF1-mediated WNT1/b-catenin signaling in proliferating mdDA progenitors and differentiating mdDA precursors as depicted in Figure 10. High levels of RSPO2/LEF1-mediated WNT1/b-catenin signaling sustain the proliferation of a subset of lFP progenitors through activation of cell cycle-promoting target genes [such as *Ccnd1*, (Omodei et al., 2008)] and specify them to LMX1A^+^ mdDA progenitors, by directly activating *Lmx1a* expression in these cells. Simultaneously, high RSPO2/LEF1 levels suppress the precocious differentiation of these cells into PITX3^+^ mdDA precursors through the repression of the *Pitx3* promoter (Figures 2, 6, 7). *In vivo* support for this part of our model comes firstly, from the fact that the ∼50% WNT/b-catenin-responding and LMX1A^+^ mdDA progenitors in the *BAT-gal^+^* VM correlate well with the ∼50% reduction of mitotic cell numbers in the VM of conditional *Ctnnb1* mouse mutants (Tang et al., 2009). Secondly, because the numbers of proliferating cells in the VM of conditional mouse mutants expressing a constitutively active (stabilized) b-catenin show a corresponding increase (Joksimovic et al., 2009; Tang et al., 2010; Nouri et al., 2015). Thirdly, because the ectopic *Nurr1*^+^ mdDA precursors are preceded by the ectopic transcription of *Lef1* and *Lmx1a* in the *En1*^+/*Wnt1*^ rostral hindbrain FP progenitors. Medial FP progenitors, by contrast, do not appear to respond extensively to “canonical” WNT/b-catenin signaling and do not express *Lef1*. WNT/b-catenin-responding lFP progenitors also downregulate *Rspo2* and *Lef1* transcription once they exit the cell cycle and initiate their outward migration from the VZ/SVZ to the IZ. Additionally, WNT/b-catenin signaling might be inhibited in these cells by *Apcdd1* (Shimomura et al., 2010), which is expressed in the IZ of the murine VM (Figures 1H–H”). Under these conditions, the *Pitx3* promoter is relieved from its LEF1-mediated repression and can now be activated by other TFs, such as LMX1A (Figure 7; Chung et al., 2009) and/or PBX1 (Villaescusa et al., 2016), expressed in this region. Thereby, postmitotic PITX3^+^ mdDA precursors initiate their differentiation into mature (TH^+^) mdDA neurons while transiting toward the MZ. This part of our model is supported *in vivo* by the apparently slight de-repression of PITX3 expression in WNT/b-catenin-responding (b-GAL^+^) cells in the mediocaudal *Lef1*^–/–^; *BAT-gal^+^* VM (Figure 4). Such a b-catenin-mediated repressor function of LEF1/TCFs on vertebrate promoters has been reported (Kim et al., 2017) and might also explain the absence of an mdDA phenotype in *Lef1*^–/–^ mice (Figure 4). The lack of LEF1 would only lead to a precocious activation of the *Pitx3* promoter and differentiation of proliferating mdDA progenitors in the presence of an activating TF complex (e.g., LMX1A and/or PBX1). This seems rather unlikely, because the expression of *Pbx1* is also restricted to postmitotic mdDA precursors in the IZ/MZ (Figures 1I–I”; Villaescusa et al., 2016). On the other hand, the loss of inhibitory *Lef1* might be compensated by the b-catenin-independent transcriptional repressor *Tcf7l1* (Mao and Byers, 2011), another LEF1/TCF TF expressed in the mouse VM whose transcription was unaffected in the *Lef1*^–/–^ mutants (Figure 4). This assumption is also supported by the unchanged b-GAL expression pattern in the midbrain of E12.5 *Lef1*^–/–^; *BAT-gal^+^* embryos (Figure 4). Yet most importantly, our model can account for the somewhat paradoxical finding that high levels of WNT1/b-catenin signaling, achieved by *Wnt1* overexpression, treatment with high WNT1 concentrations or b-catenin stabilization in mutant mice or differentiating murine PSCs, lead to a reduced number of mdDA neurons despite an increased mdDA progenitor proliferation (Joksimovic et al., 2009; Tang et al., 2010; Joksimovic et al., 2012; Fukusumi et al., 2015; Nouri et al., 2015).

### WNT/b-Catenin-Responsiveness Is Restricted to an mdDA Progenitor Subset in the Mouse

The restricted expression of *Lef1*/LEF1 in the lFP of the mediocaudal VM and the unchanged proportion of ∼50% b-GAL^+^/LMX1A^+^ and ∼35% b-GAL^+^/PITX3^+^ double-positive cells in the *BAT-gal^+^* embryos strongly suggest that only a subset of the LMX1A^+^ mdDA progenitors and PITX3^+^ mdDA precursors/neurons respond to WNT/b-catenin signaling throughout prenatal development in the mouse. The detection of invariably non-WNT/b-catenin-responding mdDA precursors in the homozygote *Wnt1*^–/–^; *BAT-gal^+^* VM supports this view and suggests that WNT1 is the main ligand activating the WNT/b-catenin pathway in the murine FP. A previous study has shown by genetic inducible fate-mapping that *Wnt1*-expressing cells contribute to the three major mdDA subpopulations (SNc, VTA and retrorubral field) in the adult mouse brain, but it remained unknown whether these cells were also responding to WNT1/b-catenin signaling (Brown et al., 2011). The percentage of *Wnt1*-derived mdDA neurons in that study was lower (∼15% or less) than the proportion of WNT/b-catenin-responding mdDA precursors/neurons in our study (b-GAL^+^ cells: ≥32%). Thus, it is very likely that the subset of WNT/b-catenin-responding mdDA progenitors not only comprises *Wnt1*-expressing cells. Moreover, the *Wnt1*-lineage stopped to contribute to these neurons after E13.5 (Brown et al., 2011). This strongly suggests that the b-GAL^+^ mdDA cells in the E14.5/17.5 *BAT-gal^+^* VM either respond to “canonical” WNT ligands other than WNT1, or represent cells that have already ceased to respond to a WNT/b-catenin signal but remain b-GAL^+^ due to the perdurance of this protein in the cells’ nucleus. We detected only sporadic b-GAL^+^ mdDA neurons in the adult *BAT-gal^+^* VM, which supports the latter view.

The WNT/b-catenin-responding mdDA progenitors were mostly located in the SOX6-negative lFP (only ∼23% of the SOX6^+^ mFP cells were also b-GAL^+^) of the mediocaudal *BAT-gal^+^* VM. Nevertheless, the WNT/b-catenin-responding mdDA progenitors contributed to an almost equal proportion (∼27%) of SOX6^+^ and OTX2^+^ mdDA neurons at E18.5. This finding corresponds well with an equal contribution of *Wnt1*-expressing cells to postmitotic SNc and VTA DA neurons reported previously (Brown et al., 2011). However, it contradicts other studies showing that SNc DA neurons derive mostly from SOX6^+^ mFP progenitors, whereas VTA DA neurons arise preferentially from posterior lFP progenitors expressing OTX2 (Omodei et al., 2008; Blaess et al., 2011; Hayes et al., 2011; Di Giovannantonio et al., 2013; Panman et al., 2014). The generation of novel, *BAT-gal* promoter-based mouse genetic tools (inducible Cre/Flp recombinase driver mice) is thus necessary for a precise fate-mapping of the mdDA progeny from WNT/b-catenin-responding cells. The conditional inactivation of the *Ctnnb1* or *Wnt1* genes in the developing mouse VM affects more pronouncedly the generation of mature TH^+^ mdDA neurons in the VTA at intermediate and caudal levels of the mutant midbrain (Joksimovic et al., 2009; Tang et al., 2009; Yang et al., 2013). Thus, it is still possible that WNT1/b-catenin signaling is required more prominently for the development of posterior (mediocaudal, VTA) compared to anterior (rostrolateral, SNc) mdDA neurons.

Our model also suggests that additional WNT1/b-catenin signaling inhibitors, such as DKK3, are required for a further reduction of WNT/b-catenin signaling levels in the murine VM/mdDA domain, which is necessary for the proper differentiation of a WNT/b-catenin-responding mdDA progenitor subpopulation into specific mdDA neuron subsets (Fukusumi et al., 2015). These variances in the molecular programming codes during mdDA neuron development are particularly relevant for the design of robust and reproducible protocols for the differentiation of PSCs into the different mdDA neuron subsets of the mammalian brain (Arenas et al., 2015; Bissonette and Roesch, 2016).

## Data Availability Statement

The datasets presented in this study can be found in online repositories. The names of the repository/repositories and accession number(s) can be found below: https://www.ncbi.nlm.nih.gov/geo/query/acc.cgi?acc=GSE99618.

## Ethics Statement

The animal study was reviewed and approved by Institutional Animal Care and Use Committee (Committee for Animal Experiments and Laboratory Animal Facility), Helmholtz Zentrum München.

## Author Contributions

PN, SG, BR, MI, CP, and CL performed experiments. PN, SG, BR, MI, DT, CKe, JB, CB, WW, and NP analyzed data. AB, AD, EE, and CKl contributed materials and reagents. PN, SG, BR, MI, and CP contributed to initial writing of the manuscript. NP designed research and wrote the manuscript. WW and NP provided funding. All authors contributed to the article and approved the submitted version.

## Conflict of Interest

The authors declare that the research was conducted in the absence of any commercial or financial relationships that could be construed as a potential conflict of interest.
