# Supplementary material for: Dose-Dependent and Subset-Specific Regulation of Midbrain Dopaminergic Neuron Differentiation by LEF1-Mediated WNT1/b-Catenin Signaling
Source: Front Cell Dev Biol. 2020 Oct 26;8:587778. doi: 10.3389/fcell.2020.587778 (PMC7649324; doi:10.3389/fcell.2020.587778)
Supplement: Supplementary file 1 [file Data_Sheet_1.PDF]

Supplementary Material

1 Supplementary Figures and Tables

1.1 Supplementary Figures

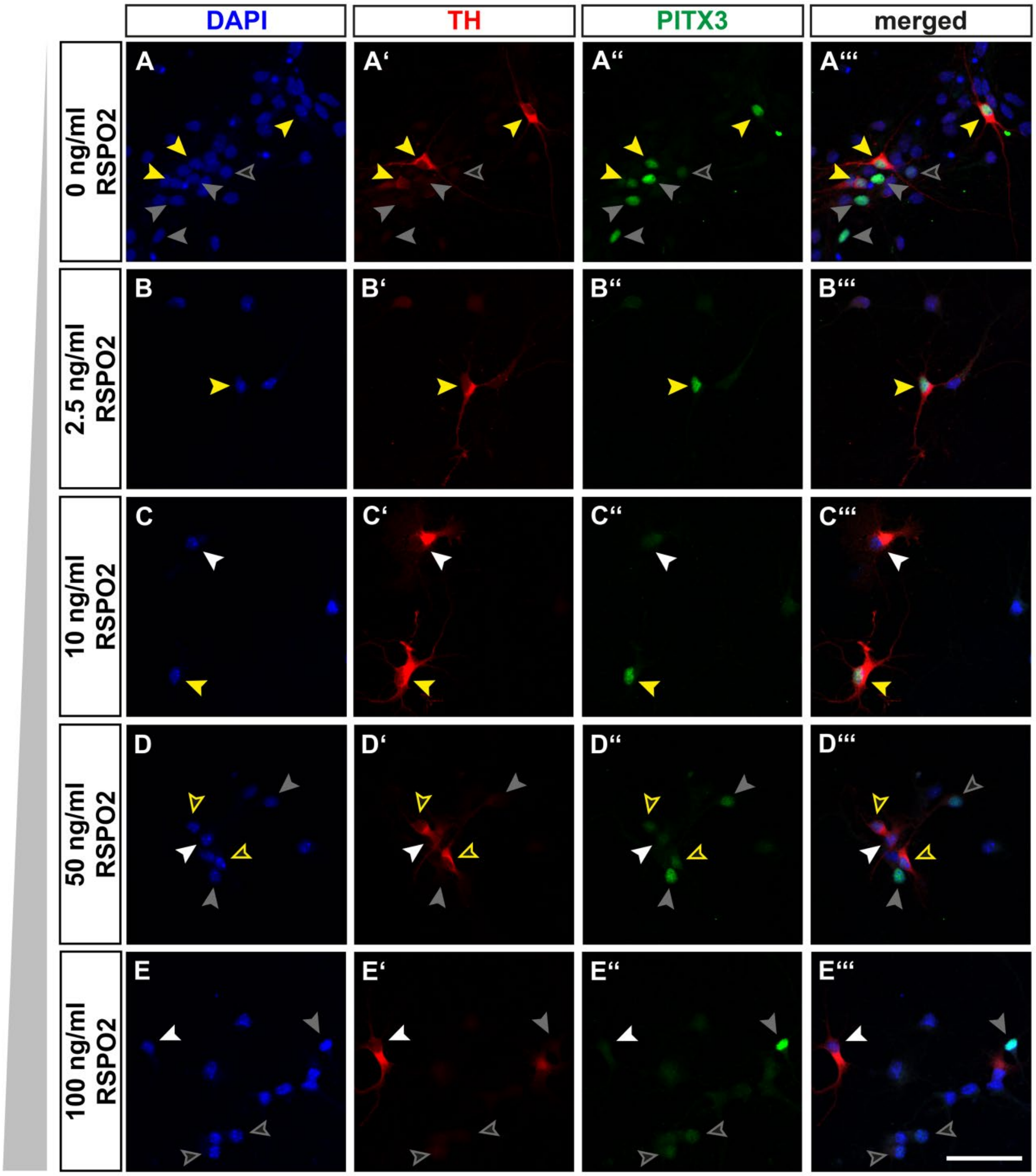

Supplementary Figure 1: RSPO2-mediated activation of WNT1/b-catenin signaling inhibits the differentiation of PITX3$^+$ mdDA neurons *in vitro*. (A-E''') Representative confocal close-up views of VM primary cells isolated from E11.5 wild-type (*CD-1*) mouse embryos and treated for 7 d (10 DIV) with increasing concentrations of RSPO2 protein. Cells were immunostained for TH (red; A'-E'), PITX3 (green; A''-E''), and counterstained with DAPI (blue; A-E). The rightmost panel (A'''-E''') depicts the merged images. Yellow arrowheads in (A-D''') point at PITX3$^+$/TH$^+$ double-positive cells; white arrowheads in (C-E''') point at PITX3$^-$/TH$^+$ single-positive cells; gray arrowheads in (A-A'''; D-E''') point at PITX3$^+$/TH$^-$ single-positive cells; and open arrowheads in (A-A'''; D-E''') point at PITX3$^+$ cells with very low immunocytochemical signal intensities. Scale bar: 50 µm (E''').

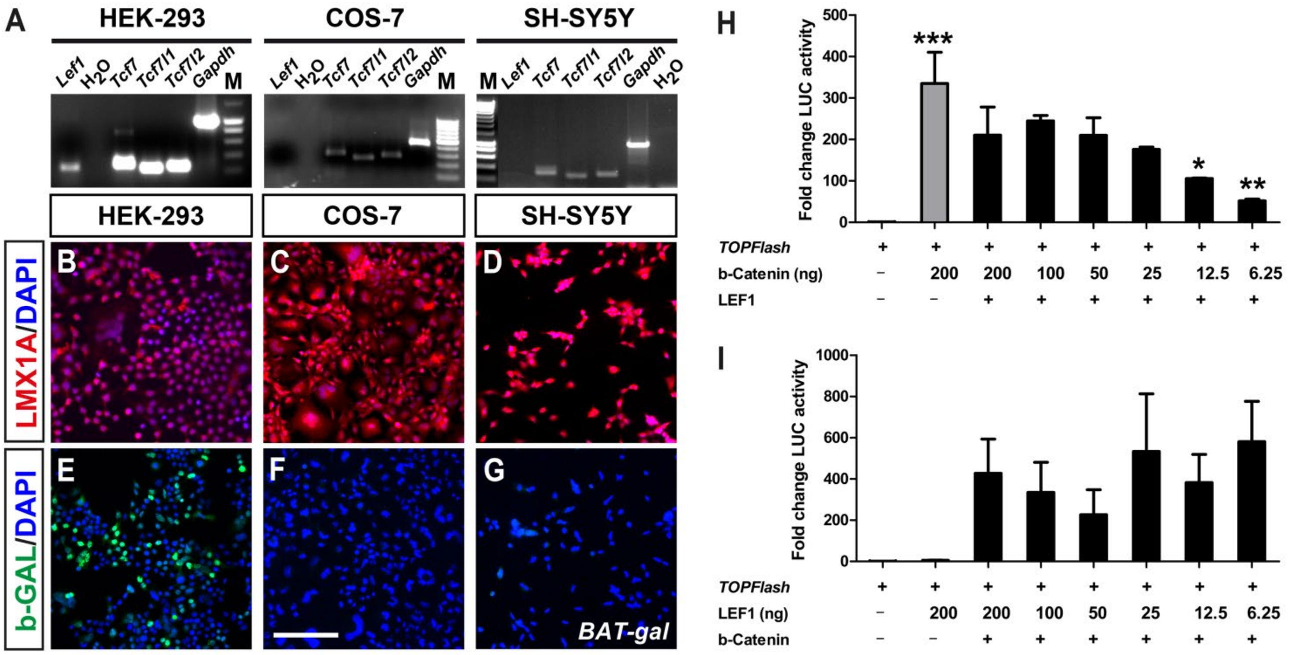

Supplementary Figure 2: Dose-dependent activation of LEF1/TCF-mediated WNT/b-catenin signaling in WNT/b-catenin-responding HEK-293 cells. (A) RT-PCR analyses of total RNA isolated from HEK-293, COS-7 and SH-SY5Y cells for the detection of *LEF1*/*Lef1*, *TCF7*/*Tcf7*, *TCF7L1*/*Tcf7l1* and *TCF7L2*/*Tcf7l2* transcription. H_2O, negative PCR control; *GAPDH*/*Gapdh* (Glyceraldehyde-3-phosphate dehydrogenase), positive PCR control; M, DNA ladder. **(B-G)** Representative confocal overviews of HEK-293 (B,E), COS-7 (C,F) and SH-SY5Y (D,G) cells, immunostained for LMX1A (red; B-D) or b-GAL after transfection of the *BAT-gal* reporter construct (green; E-G) and counterstained with DAPI (blue; B-G). **(H,I)** Fold change of luciferase (LUC) activity in HEK-293 cells (relative to only *TOPFlash*-transfected cells, set as 1) after transfection with *TOPFlash* reporter and decreasing amounts of *S33Y-b-catenin* (H) or *LEF1* cDNA (I) alone or together with *LEF1* (H) or *S33Y-b-catenin* (I) plasmids, respectively (n = 3 independent experiments; statistical testing for significance was done between the only *TOPFlash*-transfected cells and the *TOPFlash* + *S33Y-b-catenin* or *LEF1*-transfected cells (grey bars), and between the latter and cells transfected with *TOPFlash* reporter and decreasing amounts of *S33Y-b-catenin* or *LEF1* plasmids in the presence of *LEF1* or *S33Y-b-catenin* (black bars), respectively, using one-way ANOVA followed by Bonferroni's multiple comparisons *post hoc* tests; $F(7,15) = 7.1$, $P = 0.0008$). Single asterisk, $P<0.05$; double asterisks, $P<0.005$; triple asterisks, $P<0.0001$. Scale bar: 100 µm (F).

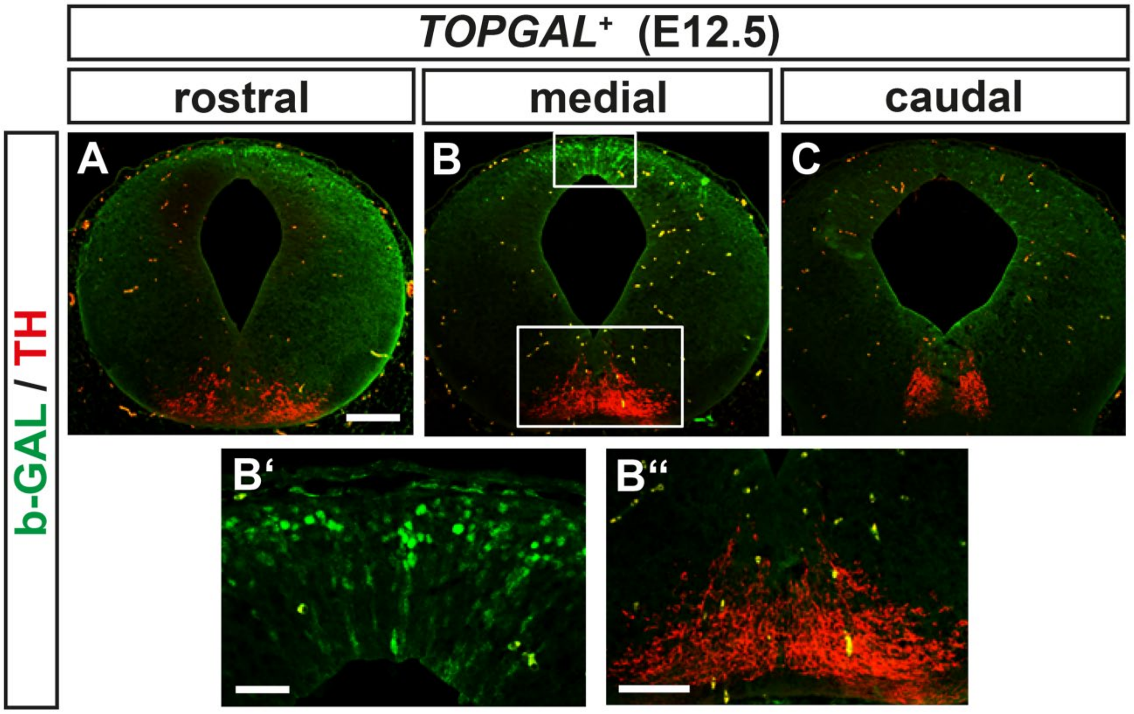

Supplementary Figure 3: The *TOPGAL* mouse is not a reporter of WNT/b-catenin signaling in the mouse VM. (A-C) Representative overviews (A-C) and close-up views of the DM (B') and VM (B'') (corresponding to the boxed areas in B) on coronal sections (dorsal top) at different rostrocaudal levels of the midbrain from a *TOPGAL* embryo at E12.5, immunostained with antibodies against b-GAL (green) and TH (red); overlapping expression domains appear in yellow. Scale bars: 200 µm (A); 100 µm (B''); 50 µm (B').

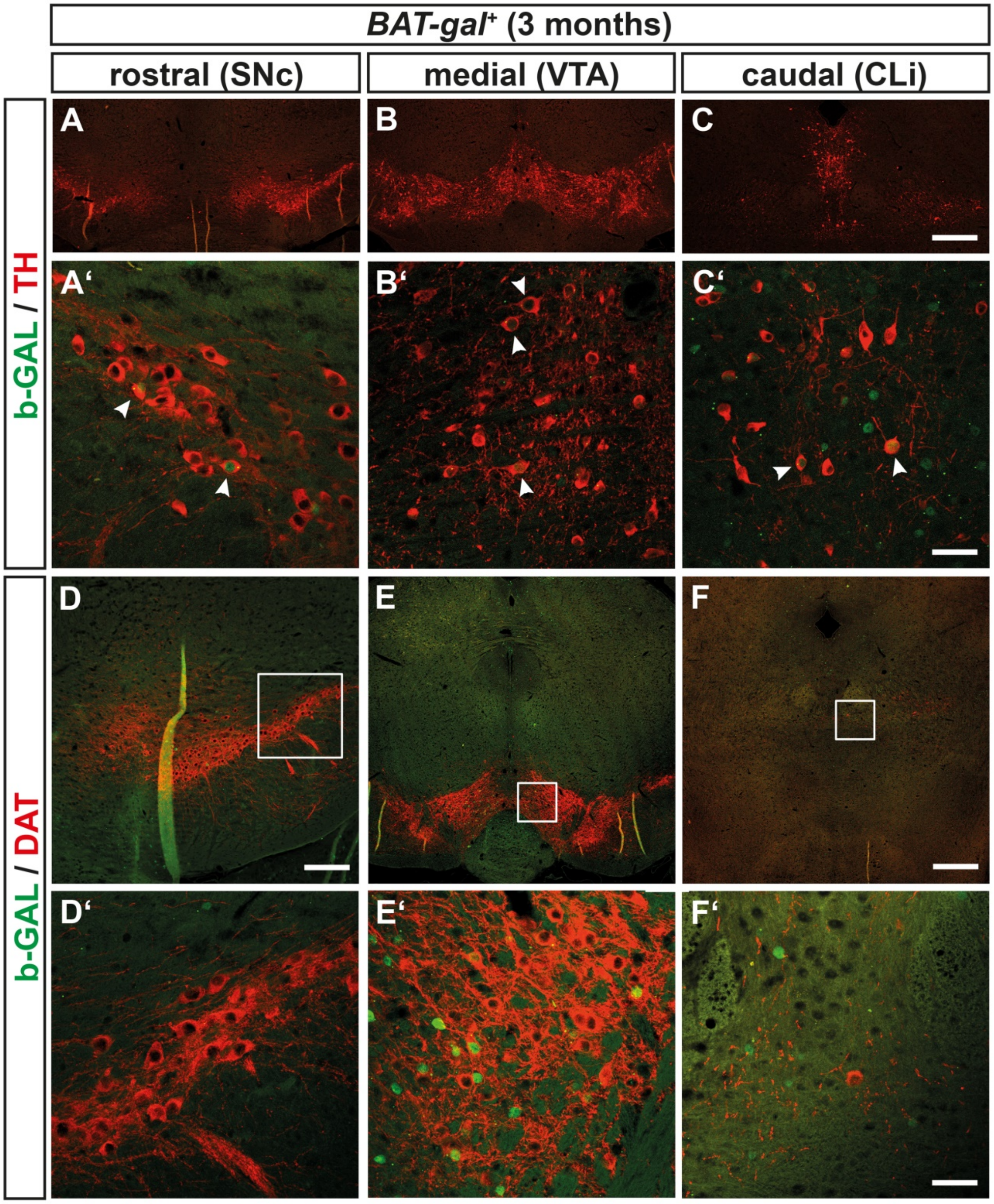

Supplementary Figure 4: mdDA neurons in the adult mouse brain are mostly non-WNT/b-catenin-responding. (A-F') Representative overviews (A-C,D-F) and close-up views of the SNc, VTA and CLi (A'-C'; D'-F', corresponding to the boxed areas in D-F, respectively) on coronal sections (dorsal top) at different rostrocaudal levels of the midbrain from 3 months-old *BAT-gal*$^{+}$ mice, immunostained with antibodies against b-GAL (green) and TH (red; A-C') or DAT (red; D-F'); overlapping expression domains appear in yellow. White arrowheads in (A'-C') point at b-GAL^{+}/TH^{+} double-positive cells. Abbreviations: CLi, caudal linear nucleus of the raphe; SNc, Substantia nigra pars compacta; VTA, ventral tegmental area. Scale bars: 250 µm (C,F); 100 µm (D); 50 µm (C',F').

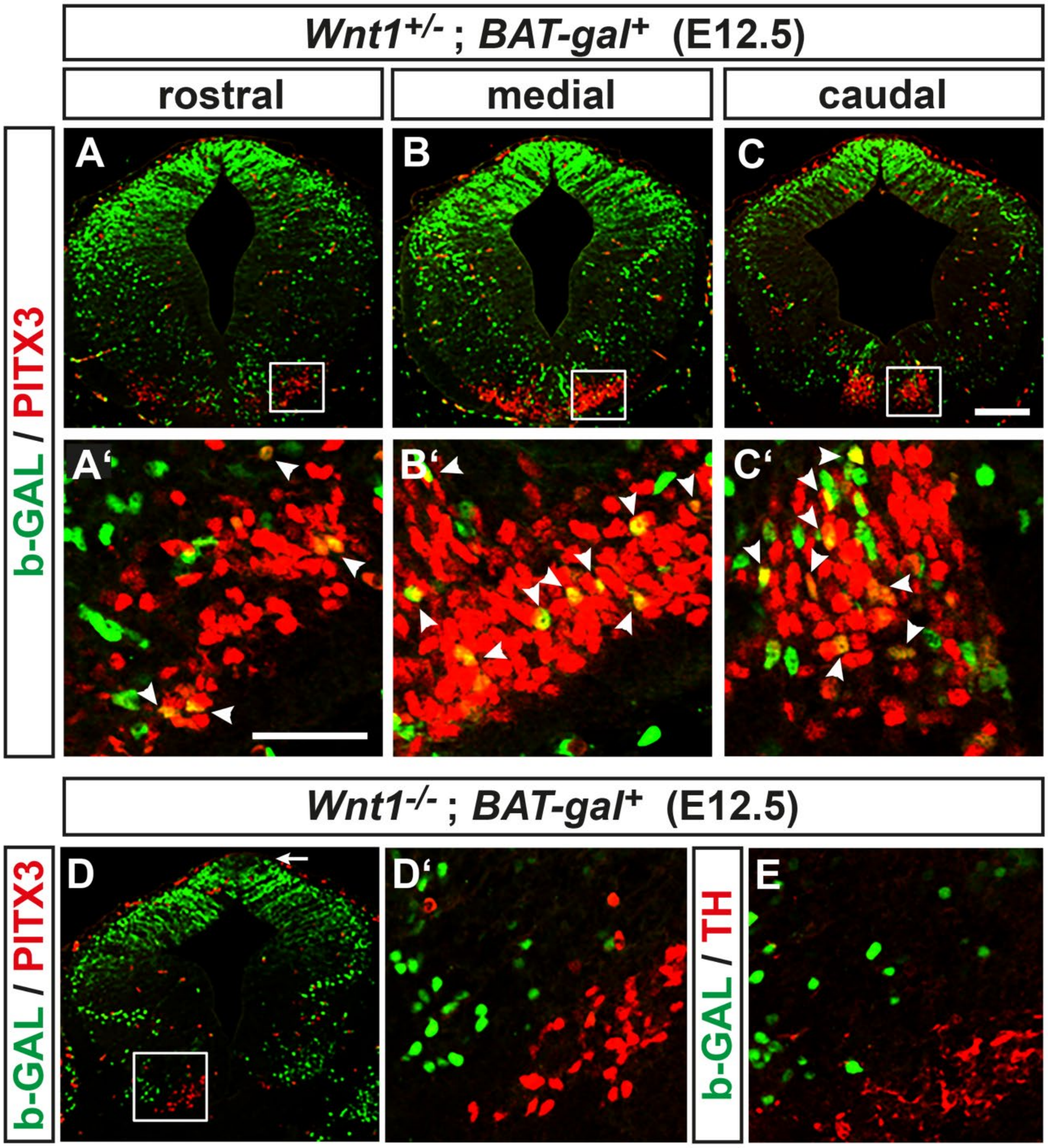

Supplementary Figure 5: *Wnt1* is the main activating WNT-ligand in the mouse FP. (A-E) Representative overviews (A-D) and close-up views of the VM (A'-D',E; corresponding to the boxed areas in A-D, respectively) on coronal sections (dorsal top) at different rostrocaudal levels of the midbrain from $Wnt1^{+/-}$; $BAT\text{-}gal^{+}$ (A-C') and $Wnt1^{-/-}$; $BAT\text{-}gal^{+}$ (D-E) mouse embryos at E12.5, immunostained with antibodies against b-GAL (green) and PITX3 (red; A-D') or TH (red; E); overlapping expression domains appear in yellow. White arrowheads in (A'-C') point at b-GAL^{+}/$PITX3^{+}$ double-positive cells. White arrow in (D) points at the b-GAL-negative roof plate. Scale bars: 200 µm (C); 50 µm (A').

1.2 Supplementary Tables

Supplementary Table 1: Primer and PCR conditions used for genotyping, ISH probe cloning, ChIP-PCR, site-directed mutagenesis of LEF1/TCF BSs/*WREs* and RT-PCR; and siRNAs

Gene (application)	Forward primer (5' → 3')<br>Reverse primer (5' → 3')<br>or mutagenic primer or siRNA (5' → 3')	Product length (bp)	Tm (°C)	Cycles
LacZ<br>(*BAT-gal* genotyping)	GGTGGCGCTGGATGGTAA<br>CGCCATTTGACCACTACC	613	60	30
LacZ<br>(*TOPGAL* genotyping)	CGTGGCCTGATTCATTCC<br>ATCCTCTGCATGGTCAGGTC	315	60	30
Lef1 wt<br>(*Lef1*$^{+/+}$ genotyping)	CCGTTTCAGTGGCACGCCCTCTCC<br>TGTCTCTCTTTCCGTGCTAGTTC	71	58	38
Lef1 ko<br>(*Lef1*$^{-/-}$ genotyping)	CCGTTTCAGTGGCACGCCCTCTCC<br>CATGGCGATGCCTGCTTGC	321	58	38
Apcdd1 (ISH probe)	TCTACCGGCCGTCCAGTTAC<br>TATCTGAGGATGCGCCAATG	814	60	34
Cck (ISH probe)	TGGACCCCAGCCATAGAA<br>GGAAACACTGCCTTCCGA	275	60	34
Fgf14 (ISH probe)	GCAACCTGGTGGATATCTTCTC<br>GAGTTTAGCTGGTTTGTCCAGG	830	63	35
Pbx1 (ISH probe)	CATGCGACTGGACAACATGC<br>CATGGGCTGACACATTGGTG	644	63	35
Rspo2 (ISH probe)	CTCATGGCGTTCTCAGCATC<br>GCCTTGCTTTGGATGTTTCC	941	63	35
Smarca1 (ISH probe)	CGGAATGCTCCCCAGTTTAG<br>CGCACACTGACAGCAAACAC	878	60	34
Sulf1 (ISH probe)	CTCTTCACTCCAGCCACACG<br>ACCAACATTGTGAGCCATGC	807	60	34
Sulf2 (ISH probe)	CTGGGACAGCTATGGGAAGG<br>GGGGGCAGGAACACTGTAAG	852	60	34
Tcf7 (ISH probe)	CTCCTTCCCCACAGAACTGC<br>GGGTGCACACTGGGTTTAGG	866	64	35
Tcf7l1 (ISH probe)	GCAAATCCAAGAGGCAGAGG<br>TCTACAGTGGTAAGGCACTGCTG	613	65	35
Pitx3 (LEF1 ChIP Primer 1)	CCTGTCCCTTGCAAACACTT<br>CCCCTCCTTTCCATCCTATC	362<br>(−1025 to −664)	60	32
Pitx3 (LEF1 ChIP Primer 2)	GGAAAGGAGGGGTGGTCTTT<br>TTGCCATCCACTGAGCAAC	412<br>(−675 to −264)	60	32
Pitx3 (LEF1 ChIP Primer 3)	AAGGCCTCAATTAGCCACAG<br>GCTAGCGGAGGAGAGAGTGA	388<br>(−406 to −19)	60	32
Pitx3 (LEF1 ChIP Primer 4)	TGGCACTGCACTGTGAGACT<br>CCCCTAGATTTCAAGGTGCTC	478<br>(−57 to +421)	60	32
Pitx3 (LEF1 ChIP Primer 5)	CCTTTACCAGAGAGCACCTTG<br>ACATTCCGTTCACCACTGCT	474<br>(+391 to +864)	60	32
Pitx3 (LEF1 ChIP Primer 6)	CAGACGATGTGTCCAGCAGT<br>TTCGAGTTTGTGAGGGGACT	396<br>(+831 to +1226)	60	32

Gene (application)	**Forward primer (5’ → 3’)**<br>**Reverse primer (5’ → 3’)**<br>**or mutagenic primer or siRNA (5’ → 3’)**	Product length (bp)	Tm (°C)	Cycles
Pitx3 WRE 1 (site-directed mutagenesis) *	tgagaggccgagtgctcacacAGCGAaagcacacaaggccagacagc<br>*wt:* tgagaggccgagtgctcacac**ctttg**aagcacacaaggccagacagc	#	#	#
Pitx3 WRE 2 (site-directed mutagenesis) *	aagatgctgctgtataccaAGCGAatttctctcgttccaaatcctgc<br>*wt:* aagatgctgctgtatacca**ctttg**atttctctcgttccaaatcctgc	#	#	#
Pitx3 WRE 3 (site-directed mutagenesis) *	tcgggtctcagctcccacaAGCGActgccgctcgtttcgccg<br>*wt:* tcgggtctcagctcccaca**ctttg**ctgccgctcgtttcgccg	#	#	#
Lef1 (RT-PCR)	AAAGAAATGAGGGCGAATGTCGTA<br>GCTGTCATTCTGGGACCTGTACCT	257	59	30
Tcf7 (RT-PCR)	CCCCTCAATGCTTTCATGCTTTAC<br>CGAATGCATTTCTTTTTCCTCCTG	283	59	30
Tcf7l1 (RT-PCR)	TACCCCTTCCTGATGATTCCAGAC<br>GGAGAAGTGGTCGTTGCTGTAGGT	288	59	30
TCF7L2 (RT-PCR)	GCATCAGGACTCCAAAAAGGAAGA<br>TTCCCATAGTTATCCCGTGCAGAC	270	59	30
GAPDH (RT-PCR)	TGAAGGTCGGAGTCAACGGATTTGGT<br>CATGTGGGCCATGAGGTCCACCAC	983	59	30
LMX1A (RT-PCR)	TGTCTGCGAGGGCTGTCAGC<br>CAGCAGCAGAAGCAGCTCAGG	270	60	35
Gapdh (RT-PCR)	ACCACAGTCCATGCCATCAC<br>TCCACCACCCTGTTGCTGTA	450	60	35
LMX1A siRNA #144	GCUUGAUGCACUUAAGUUAdTdT (sense)<br>dTdTCGAACUACGUGAAUUCAAU (antisense)	—	—	—
nt siRNA (control)	ON-TARGETplus Non-targeting siRNA #1, Cat. No. D-001810-01-05	—	—	—

Footnotes: Oligonucleotides were purchased from Metabion International AG and biomers.net GmbH, Germany. Targeting and non-targeting siRNAs were purchased from Fermentas GmbH (Thermo Fisher Scientific), Germany.

* Mutagenized nucleotides in CAPITALS, *WRE* sequence in **bold**, and predicted LEF1/TCF BS is underlined.

# According to the instructions of the manufacturer.
